# Indoor Exposure to Selected Air Pollutants in the Home Environment: A Systematic Review

**DOI:** 10.3390/ijerph17238972

**Published:** 2020-12-02

**Authors:** Sotiris Vardoulakis, Evanthia Giagloglou, Susanne Steinle, Alice Davis, Anne Sleeuwenhoek, Karen S. Galea, Ken Dixon, Joanne O. Crawford

**Affiliations:** 1 Institute of Occupational Medicine (IOM), Edinburgh EH14 4AP, UK; eva.giagloglou@iom-world.org (E.G.); susanne.steinle@iom-world.org (S.S.); alice.davis@iom-world.org (A.D.); anne.sleeuwenhoek@iom-world.org (A.S.); karen.galea@iom-world.org (K.S.G.); ken.dixon@iom-world.org (K.D.); joanne.crawford@vuw.ac.nz (J.O.C.); 2 National Centre for Epidemiology and Population Health, Research School of Population Health, Australian National University, ACT 2601 Canberra, Australia; 3 Faculty of Health, Victoria University of Wellington, 6410 Wellington, New Zealand

**Keywords:** indoor air, chemicals, particulate matter, VOC, PAH, benzene, toluene, formaldehyde, naphthalene, residential exposure, ventilation, asthma

## Abstract

(1) Background: There is increasing awareness that the quality of the indoor environment affects our health and well-being. Indoor air quality (IAQ) in particular has an impact on multiple health outcomes, including respiratory and cardiovascular illness, allergic symptoms, cancers, and premature mortality. (2) Methods: We carried out a global systematic literature review on indoor exposure to selected air pollutants associated with adverse health effects, and related household characteristics, seasonal influences and occupancy patterns. We screened records from six bibliographic databases: ABI/INFORM, Environment Abstracts, Pollution Abstracts, PubMed, ProQuest Biological and Health Professional, and Scopus. (3) Results: Information on indoor exposure levels and determinants, emission sources, and associated health effects was extracted from 141 studies from 29 countries. The most-studied pollutants were particulate matter (PM_2.5_ and PM_10_); nitrogen dioxide (NO_2_); volatile organic compounds (VOCs) including benzene, toluene, xylenes and formaldehyde; and polycyclic aromatic hydrocarbons (PAHs) including naphthalene. Identified indoor PM_2.5_ sources include smoking, cooking, heating, use of incense, candles, and insecticides, while cleaning, housework, presence of pets and movement of people were the main sources of coarse particles. Outdoor air is a major PM_2.5_ source in rooms with natural ventilation in roadside households. Major sources of NO_2_ indoors are unvented gas heaters and cookers. Predictors of indoor NO_2_ are ventilation, season, and outdoor NO_2_ levels. VOCs are emitted from a wide range of indoor and outdoor sources, including smoking, solvent use, renovations, and household products. Formaldehyde levels are higher in newer houses and in the presence of new furniture, while PAH levels are higher in smoking households. High indoor particulate matter, NO_2_ and VOC levels were typically associated with respiratory symptoms, particularly asthma symptoms in children. (4) Conclusions: Household characteristics and occupant activities play a large role in indoor exposure, particularly cigarette smoking for PM_2.5_, gas appliances for NO_2_, and household products for VOCs and PAHs. Home location near high-traffic-density roads, redecoration, and small house size contribute to high indoor air pollution. In most studies, air exchange rates are negatively associated with indoor air pollution. These findings can inform interventions aiming to improve IAQ in residential properties in a variety of settings.

## 1. Introduction

There is increasing awareness that the quality of the indoor environment can affect our health and well-being. Indoor air quality (IAQ) in particular has an impact on multiple health outcomes, including respiratory and cardiovascular illness, allergic symptoms, cancers, and premature mortality [1]. As the world is becoming increasingly urbanised, with urban residents typically spending over 90% of their time indoors [2,3], it is important to characterise IAQ and understand which pollution sources, housing characteristics, and occupancy patterns have the largest impact on our exposure to pollutants present in the home environment.

Exposure to high concentrations of air pollutants indoors can cause both acute and chronic health effects. Examples of acute effects include exacerbation of allergic symptoms, such as atopic dermatitis, rhinitis, conjunctivitis and hay fever, and intoxication and death due to short-term exposure to very high concentrations of carbon monoxide (CO) [4]. Examples of chronic health effects include cancer and non-cancer effects associated with organic chemicals [5], respiratory effects related to second-hand tobacco smoke (e.g., chronic obstructive pulmonary disease (COPD)) [6], increased susceptibility to respiratory infections, and cardiovascular disease [7]. Certain pollutants, such as tobacco smoke and other combustion products, may aggravate asthma symptoms [8], while formaldehyde and other volatile organic compounds (VOCs) have been associated with the sick building syndrome (SBS) [9].

IAQ is a complex function of outdoor and indoor sources of pollution, environmental conditions, housing characteristics, and behavioural factors [1]. Outdoor air pollution concentrations associated with anthropogenic and natural sources, such as road traffic, wildfire smoke and re-suspension of dust, can affect indoor air pollutant levels. In addition, dispersion characteristics of pollutants surrounding the building influenced by, e.g., the type, position and distance of pollutant sources; size, shape, orientation and arrangement of buildings; topography and meteorological conditions also play a role [10,11,12,13].

Furthermore, IAQ levels are highly variable over time, depending on a range of internal factors that include (i) the physical and chemical properties of pollutants (gaseous or particulate, reactivity, deposition, size for particulates); (ii) use of household products, such as cleaning products, cosmetics and insecticides, and appliances, such as gas cookers and stoves, and building and furnishing materials such as chemical flame retardants; (iii) building characteristics including infiltration and ventilation rates; (iv) occupant behaviour and activities, e.g., opening of windows, tobacco smoking, cleaning, cooking, and use of extractor fans [14,15].

The aim of this review is to characterise air pollution exposure levels and associated health effects in homes around the world and provide evidence-based recommendations for improving IAQ. To achieve this, we carried out a systematic search and review of the evidence in relation to IAQ and human exposure in domestic environments. This review focused on measured concentrations of common chemical pollutants from a wide range of outdoor and indoor sources, related health effects, building characteristics, and locational, seasonal and occupancy patterns.

## 2. Methods

### 2.1. Literature Searches

Peer-reviewed articles published between 2000 and 2017 were included in our searches. We excluded studies published prior to 2000, as these were largely covered by a comprehensive review on IAQ and health by Jones (1999) [9], and excluded studies published in languages other than English or if their main focus was on the evaluation of outdoor air pollution or household air pollution from biomass burning in rural settings. We also excluded publications related to asbestos, radon, methane, biological pollutants, and acute carbon monoxide exposure. Furthermore, we focused on measured exposures to chemical air pollutants and particles in the domestic indoor environment, excluding studies that relied entirely on IAQ modelling. Although we extracted health information, a comprehensive review of the health effects of specific indoor pollutants (e.g., including epidemiological and toxicological studies) was beyond the scope of this review.

The following online bibliographic databases were used: ABI/INFORM, Environment Abstracts, Pollution Abstracts, PubMed, ProQuest Biological and Health Professional, and Scopus. The search results from the databases were stored in the online reference manager RefWorks. Additional manual searches in the reference lists of review papers were carried out. The search strategy adopted, including all search terms used in bibliographic database searches, is documented in Supplementary Materials.

### 2.2. Paper Screening and Data Extraction

After the searches were completed, the titles and, where available, abstracts were screened to identify studies of relevance in accordance with the inclusion criteria. In all cases, a conservative strategy was adopted, where, if the relevance or otherwise of a paper was not apparent from the title/abstract, the paper was retained for full-text scanning. Following the screening of the titles and abstracts, copies of the full papers were obtained for those included. A data extraction template was developed and trialled before being applied to the included papers. The final data extraction template included publication title, authors, pollutant(s), country, location, study design, indoor sources, occupancy patterns, dwelling characteristics, exposure levels, method(s) of measurement, number and duration of measurements, and health effects (if reported). Title, abstract and full-text screening, and data extraction for each eligible paper were undertaken independently by two reviewers, with disagreements resolved by a third reviewer. From the data extracted, summary tables were prepared to aid the assessment process, and a narrative synthesis of the evidence was carried out.

## 3. Results

**Figure 1:**
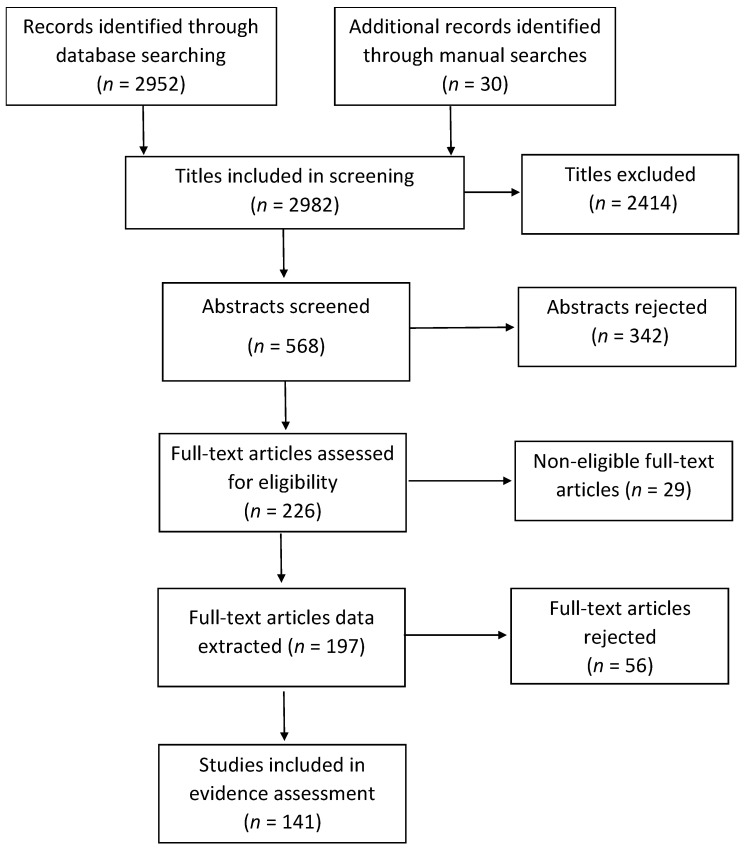
Numbers of papers at each stage of the review process (PRISMA diagram).

**Figure 2:**
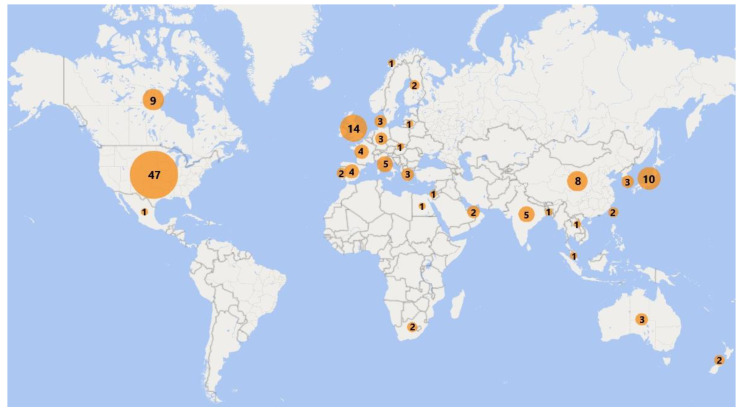
Number of IAQ studies included per country. Studies with IAQ data from multiple countries have been counted separately for each country.

**Figure 3:**
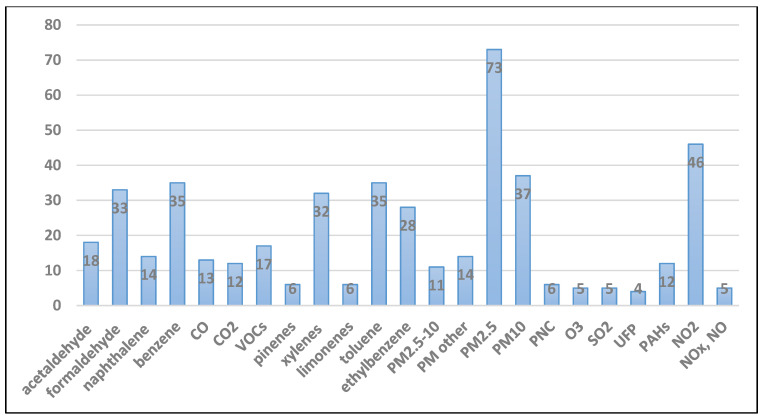
Number of studies included for each indoor pollutant. Studies reporting indoor concentrations of multiple pollutants have been counted separately for each pollutant. (PM_2.5_: particles with aerodynamic diameter < 2.5 μm; PM_10_: particles with aerodynamic diameter < 10 μm; PNC: particle number concentration; UFP: ultrafine particles.)

Overall, 2982 titles were identified and screened, 226 full-text articles were obtained and assessed for eligibility, with 141 of them included in the evidence synthesis (Figure 1). Studies from 29 countries were included, with a relatively high number of studies from the USA, UK, Japan, Canada, and China (Figure 2). IAQ studies were very sparse for the Middle East, Central Asia, Latin America and Africa. The most-studied pollutants were particulate matter (PM) as PM_2.5_ and PM_10_; nitrogen dioxide (NO_2_); volatile organic compounds (VOCs) including formaldehyde, acetaldehyde, benzene, ethylbenzene, toluene, and xylenes; and to a lesser extent polycyclic aromatic hydrocarbons (PAHs) including naphthalene; and carbon monoxide (CO) (Figure 3). Fewer studies focused on other individual VOCs (limonenes, pinenes), sulphur oxides (SO_x_), and ozone (O_3_). Carbon dioxide (CO_2_) levels were reported as an indicator of indoor ventilation in a number of studies.

A full list of the extracted information with a summary of the studies included can be found in Supplementary Materials (Table S1). Sources, exposure levels and health effects of selected chemicals and groups of pollutants are discussed in Section 3.1, Section 3.2, Section 3.3, Section 3.4, Section 3.5, Section 3.6 and Section 3.7. Housing characteristics, occupancy, ventilation and activity patterns, seasonal and climatic influences, and dwelling location, setting and within-building variability are discussed in Section 4.1, Section 4.2, Section 4.3 and Section 4.4.

### 3.1. Particulate Matter

#### 3.1.1. Exposure Levels, Sources and Determinants

Particulate matter (PM) is a complex mixture of organic and inorganic chemicals, including organic carbon (OC) and elemental carbon (EC), which vary by season and geographic location [16,17]. Indoor particles comprised ambient particles of different size fractions (typically reported as PM_10_, PM_2.5_, and UFP) that infiltrated from outdoors, and particles that were generated indoors [18,19]. Major indoor PM sources included smoking, cooking (particularly using kerosene and biomass fuels), wood stoves and furnaces, use of incense and candles for fine particles (PM_2.5_), and cleaning, presence of pets, and people’s movements for coarse particles [20,21,22,23,24,25,26].

Seventy-three studies reported indoor PM_2.5_, with mean concentrations ranging between 1.7 μg/m^3^ in Quebec City, Canada [27], and 428.6 μg/m^3^ in homes with hookah (i.e., water pipe) smoking in Dubai, UAE [28] (Table S1). Cooking and smoking were major indoor sources of PM_2.5_ in homes in the UK [29,30] and USA [31]. In addition to indoor smoking [32,33], PM_2.5_ levels were strongly influenced by the use of coal for cooking [34] and motor vehicle emissions [35] in China. The use of kerosene and biomass fuels for cooking increased indoor PM_2.5_ concentrations to a level 7-fold greater than the World Health Organization (WHO) guidelines in Dhaka, Bangladesh [36].

Thirty-seven studies reported indoor PM_10_, with mean concentrations ranging between < 11.0 in Quebec City, Canada [37], and 1275 μg/m^3^ in houses with soil floor in Lao PDR [38]. Very high PM_10_ levels were also reported in Delhi and Agra, India [39,40]. Relatively high indoor PM_10_ in southern Europe compared to northern and central Europe was possibly due to higher ingress of mineral dust from outdoor sources [41]. Higher indoor PM_10_ was reported in Portuguese [41] and UK homes [42] compared to outdoor levels, although outdoor sources were a significant contributor to indoor concentrations. OC content of PM_10_ was higher indoors than outdoors showing the influence of indoor sources such as smoking, cooking, biomass burning and movement of people, while EC sources were mainly outdoors [41].

Indoor UFP concentrations were affected by outdoor UFP concentrations, building characteristics and infiltration, air exchange rates (AERs), indoor and outdoor meteorological parameters, personal behaviours, as well as indoor sources and human activities such as cooking, candle burning, heating devices, environmental tobacco smoke (ETS), and office equipment [43,44,45]. In a study in California, USA, cooking caused the highest indoor UFP exposures [46].

#### 3.1.2. Health Effects

Exposure to ambient PM has been linked to decline in pulmonary function and cardiovascular events possibly through inflammation. Indoor UFP exposure is of particular concern due to the enhanced ability of UFP to cause oxidative stress and inflammation in the lungs [47]. However, less is known about individual exposure to UFP inside and outside modern homes and associated health effects [48].

Higher concentrations of indoor (fine and coarse) PM were associated with increases in asthma symptoms and relief medication use in children in Baltimore, USA [49,50]. Significantly positive associations were found between indoor PM_2.5_ and new wheeze in children in New York City, USA [51]. However, a study in California, USA, showed mixed respiratory effects of PM in children with asthma in various microenvironments [52]. Indoor exposure to PM increases the risk of acute lower respiratory tract infections, which are the leading cause of death in young children in low-income countries [36]. Increased indoor PM levels have been associated with worse respiratory symptoms and increased risk of exacerbation in patients with COPD [53,54].

### 3.2. Nitrogen Dioxide

#### 3.2.1. Exposure Levels, Sources and Determinants

NO_2_ is a by-product of combustion produced by motor vehicles, energy generation and other outdoor sources involving combustion, as well as indoor sources such as gas appliances and kerosene heaters (e.g., [55,56]).

Indoor NO_2_ levels reported in forty-six studies ranged between 3.4 μg/m^3^ in homes in Quebec City, Canada [27], and 1210 μg/m^3^ in houses with indoor smoking and/or cooking in Lao PDR [38], in cases exceeding the WHO guidelines [1] for acute (200 μg/m^3^) or chronic (40 μg/m^3^) exposure (Table S1). Indoor levels where NO_2_ sources, such as gas appliances, were present were much higher than outdoors, where the primary source of NO_2_ was road traffic [57]. NO_2_ concentrations were higher in all rooms in houses in the UK [58] and Germany [59] where gas cookers were used. NO_2_ levels in kitchens with a gas cooker in the UK were twice as high as in those with an electric cooker during winter [58]. Nitrogen oxides (NO_x_) and NO_2_ were higher in homes with cooktop pilot burners, relative to gas cooking without pilots. In homes in California, USA, where residents cooked 4 h or more per day with gas, self-reported use of kitchen exhaust fans was associated with lower NO_x_ and NO_2_ exposure [60].

Seasonal effects were also identified with increased indoor concentrations of NO_2_ in winter in Japan [55], which was thought to be due to the use of gas heaters. Cyrys et al. [59] observed outdoor NO_2_ concentrations approximately twice as high as indoor levels in Germany, while Cibella et al. [61] and Zipprich et al. [62] observed significantly higher indoor NO_2_ concentrations than outdoor concentrations in Italy and USA respectively. Overall, the most important predictors of indoor NO_2_ concentrations were gas cooker use, followed by ventilation and outdoor NO_2_ levels.

#### 3.2.2. Health Effects

Most reviewed evidence, mainly from US studies, suggested that exposure to higher indoor NO_2_ concentrations leads to symptoms in children with asthma, including chest tightness, shortness of breath, wheeze, cough, nocturnal symptoms, increased number of asthma attacks and inhaler use, and decreased forced expiratory volume in one second (FEV1) [63,64,65,66,67]. Children living in inner cities appeared to be at higher risk for the adverse effects of NO_2_ given their relatively high indoor exposure [64], although increased risk of asthma morbidity also occurred at NO_2_ concentrations common in urban and suburban homes [57]. In addition, exposure to higher indoor NO_2_ concentrations was associated with increased respiratory symptoms and risk of COPD exacerbations in former smokers with moderate to severe COPD [56].

### 3.3. Volatile Organic Compounds

#### 3.3.1. Exposure Levels, Sources and Determinants

VOCs are emitted from a very wide range of indoor and outdoor sources through combustion and evaporation, e.g., cigarette smoking, solvent-related emissions, renovations, household products and pesticides [5,68]. Typical VOCs found in the indoor environment include benzene, toluene, ethylbenzene and xylenes (BTEX) from fuel combustion and evaporation, and house renovations; benzene and styrene from cigarette smoking; alkanes from natural gas; 1,4-dichlorobenzene from moth repellents; a-pinene from wood-based building materials; and limonene from fragranced household cleaning and laundry products (e.g., [69,70,71,72]).

Forty-two studies reported indoor concentrations of one or more VOCs (excluding studies that only reported formaldehyde or other carbonyls). VOC levels in homes depended on many factors, such as the strength of emission sources, ventilation rates, and the indoor oxidative environment, which reflected differences in chemical use, building design and materials, occupant behaviour, and season (e.g., [32,73,74]). Reported VOC concentrations were generally higher indoors than outdoors, including for benzene, particularly in colder seasons due to reduced ventilation and the use of oil and gas heaters [75,76,77,78,79,80,81]. Indoor sources were dominant for most VOCs and particularly for limonene, a-pinene, hexanal, pentanal, o-xylene, and n-dodecane [76,82]. Use of artificial air freshener was significantly associated with total VOC (TVOC), benzene, toluene and ethylbenzene [78].

Thirty-nine studies reported indoor levels of benzene and/or toluene, ranging from 0.6 and 3.0 µg/m^3^ in Kaunas, Lithuania [75], to 24.8 μg/m^3^ in Perth, Australia [74], and 325.5 µg/m^3^ in Sapporo, Japan [83], respectively (Table S1). Studies previously reviewed by Sarigiannis et al. (2011) reported similar, though narrower, ranges of benzene (1.2–17.0 μg/m^3^) and toluene (4.3–86.2 μg/m^3^) in homes within the European Union [5]

Homes where ETS was present had higher concentrations of almost all VOCs including benzene [70,72,76,84,85]. Indoor toluene, ethylbenzene and xylenes concentrations were mainly dominated by outdoor sources such as road traffic [75,77], although toluene and o-xylene concentrations were also elevated in smoking homes [85] and toluene levels were affected by the presence of carpets [76]. Elevated levels of benzene and toluene were also found in basements and garages adjacent to residences, probably due to the use and storage of solvents, petrol and petrol-powered equipment [70,82].

Use of low-emission and non-absorbent indoor materials, and climatic conditions that favour natural ventilation, resulted in reduced indoor benzene, toluene and xylene levels in Athens, Greece, and in Seoul, Korea [77,78]. However, two studies from northern Europe reported indoor concentrations of toluene and xylene significantly higher than outdoor levels [86,87]. An international study showed that levels of VOCs in new homes decreased dramatically and were close to the mean values for the older homes after one year from construction [88]. However, a study from Hong Kong [89] found no relationship between VOC concentrations (other than formaldehyde) and building age.

Occupant density was positively associated with indoor VOC and BTEX concentrations in Australia [71] and Canada [70], respectively. Indoor VOC concentrations were negatively correlated with ventilation. Indoor alkanes and aromatics were associated with proximity to major roads. Levels of VOCs in Australian dwellings were lower than those from studies in North America and Europe, probably due to the leakier nature of Australian dwellings [71].

#### 3.3.2. Health Effects

Many VOCs are classified as known or possible carcinogens (benzene in particular is a known human carcinogen mainly associated with leukaemia risk), irritants and toxicants, and measurement of TVOCs may underestimate the risks associated with individual compounds [5,70]. Most VOCs have also been reported to be significant risk factors for asthma [73,74,90,91,92], with the strongest association with benzene followed by ethylbenzene and toluene. However, after adjustment for confounding factors, no significant associations were found between residential benzene exposure and respiratory health in infants in Spain [79], or between low-level exposure to VOCs (except for d-limonene) and asthma status in children in Portugal [93]. Exposure to high concentration of VOCs during infancy increased the risk of atopic dermatitis in Korean children [94]. Residential exposure to a-pinene was associated with throat and respiratory symptoms in Japan [83]. However, there were no significant effects of a-pinene on SBS symptoms [95]. Overall, there was less attention on the SBS in the studies reviewed here than in the earlier IAQ review by Jones (1999) [9].

### 3.4. Formaldehyde and Other Carbonyls

#### 3.4.1. Exposure Levels, Sources and Determinants

Formaldehyde is mainly emitted from building materials (e.g., insulating materials and pressed-wood products), household products (e.g., paints, cleaning products, pesticides, adhesives), parquet flooring and carpets, smoking (although not a dominant source), and unvented fuel-burning appliances (e.g., [5,55,89,96]). Carbonyl compounds can also occur in the indoor environment as secondary pollutants as products of the reaction of a primarily pollutants with ozone.

Thirty-three studies reported indoor formaldehyde levels with concentrations ranging from 7.5 µg/m^3^ in Quebec City, Canada [37], to 134 µg/m^3^ in new homes (first year) across various cities in Japan [88] (Table S1). Studies previously reviewed by Sarigiannis et al. (2011) reported a narrower range of formaldehyde concentrations (12.3–46.1 μg/m^3^) in homes within the European Union [5]. Differences among indoor concentrations of formaldehyde were due to differences in building ages, geography, building materials, furniture, and household products. Formaldehyde levels were generally higher in newer houses [55,78,89], particularly in those with wooden frames or furniture bought new or restored [76,97]. Maruo et al. (2010) estimated the relationship between formaldehyde levels and the age and temperature of homes in Japan [98]. They obtained the highest formaldehyde concentrations for apartments 0–2 years after their renovation, with a linear relationship between formaldehyde concentration and years after renovation. Formaldehyde and a-pinene related to wooden materials needed a longer flushing period than other VOCs in new homes [88].

Indoor formaldehyde and styrene levels in Hong Kong were higher than in other East Asian cities in Japan, China, Korea, Hong Kong and Taiwan, reflecting the higher prevalence of household products and materials containing these chemicals in Hong Kong [89]. An Italian study [97] reported lower indoor concentrations of formaldehyde than those reported in Japan [99] and France [96,100]. Formaldehyde levels ranged very widely in households in England [101] and Canada [102,103].

Indoor sources were dominant for formaldehyde, acetaldehyde and acetone [76,104]. Formaldehyde concentrations tended to be higher in summer when temperatures were higher. For example, Rancière et al. [105] found an increase in the concentrations of formaldehyde as a result of indoor chemistry involving oxidants such as ozone, which is present at higher concentrations in summer, and unsaturated organic compounds, such as terpenes, in homes in Paris. However, formaldehyde and acetaldehyde levels were significantly higher in winter than in spring-summer in dwellings in Bari, Italy [97], possibly because of windows kept open during good weather.

#### 3.4.2. Health Effects

Formaldehyde is carcinogenic to humans, based on increased risk of nasopharyngeal cancer and leukaemia [1]. It is also an irritant of the upper respiratory tract with symptoms such as eye, nose and throat irritation commonly associated with indoor exposure. Exposure to low concentrations of formaldehyde for a short period of time influences the skin barrier function in patients with atopic dermatitis. Low level exposure to indoor formaldehyde may increase the risk of allergic sensitization to common aeroallergens in children [78], although this risk was low in households in south Italy [97]. Young children experiencing recent indoor renovation in German houses showed increased risk of eczema [106]. Formaldehyde was associated with neurological symptoms (difficulty concentrating) in a study in UAE [107]. Elevated levels of indoor aldehydes increased the possible risk of SBS in residents living in new houses in Japan [108].

### 3.5. Polycyclic Aromatic Hydrocarbons

#### 3.5.1. Exposure Levels, Sources and Determinants

PAHs are products of incomplete combustion generated through wood, coal, oil, and gas burning, smoking, waste incineration, industrial power generation, and vehicle emissions (e.g., [75]). PAHs with low molecular weight (i.e., with 2 or 3 rings) are emitted in the gaseous phase, while high-molecular-weight PAHs (i.e., with 5 or more rings) are emitted in the particulate phase [109].

Twelve studies reported indoor levels of PAHs, with the sum of different groups of PAHs ranging from 1.5 ng/m^3^ in homes in Agra, India [40], to 9568 ng/m^3^ in homes in Hangzhou, China [110] (Table S1). A number of studies, mostly from China and Japan, have shown residential indoor PAH concentrations generally higher than outdoor [110,111], but the opposite was observed in a suburban home in Brisbane, Australia [112]. Indoor air PAHs, especially low-molecular-weight PAHs, mainly came from indoor emission sources. Higher PAH concentrations were observed in smoking houses in China, Japan, and the UK [84,113]. In non-smoking houses, moth repellent and cooking practice were the main sources of 2- and 3-ring PAHs, respectively. Low-molecular-weight PAHs were also associated with kerosene heating and the outdoor pollution in non-smoking houses. In a study in Italy, the presence of fireplaces could explain the higher indoor levels of total PAHs and pyrene (4-ring PAHs) found in rural households compared to urban households [114]. High-molecular-weight (5- and 6-ring) PAHs in indoor air were mainly associated with outdoor sources and their levels tended to be the same or lower than those in outdoor air.

PAH concentrations both indoor and outdoor were significantly higher in the gaseous fraction (2-, 3-, and 4-ring PAHs) than in the particulate fraction (5- and 6-ring PAHs) in India (Masih et al., 2010). PAHs in the indoor environment were mainly attributable to gas usage, cooking (frying and oil combustion), smoking and incense burning, whereas outdoors the most common sources of PAHs were petrol and diesel vehicles.

#### 3.5.2. Health Effects

There is evidence for the genotoxicity and carcinogenicity of many PAHs in animal species, and epidemiological studies demonstrated that there is a correlation between PAH exposure and cancer incidence for various human tissues [115].

### 3.6. Naphthalene

#### 3.6.1. Exposure Levels, Sources and Determinants

Naphthalene (both a VOC and a 2-ring PAH) is a ubiquitous pollutant, and very high concentrations are sometimes encountered indoors when it is used as an insect repellent or deodorant [82,116]. Other sources that have an impact on indoor levels include (to lesser extents) cigarette smoking and motor vehicle emissions.

Fourteen studies reported naphthalene levels in domestic indoor environments, with concentrations ranging from 0.12 μg/m^3^ in homes in Kaunas, Lithuania [75], to 26.3 μg/m^3^ (and in one case exceeding 1000 μg/m^3^) in houses in Michigan, USA [82]. Jia and Batterman (2010) suggested typical naphthalene concentrations ranged from 0.18 to 1.7 μg/m^3^ in non-smokers’ homes [117], which were less variable than the indoor concentrations reported in this review (Table S1). Outdoor concentrations in urban areas reported by Jia and Batterman (2010) were typically lower than indoor levels [117]. Naphthalene was the dominant PAH at both urban background and roadside locations (indoors and outdoors) in Agra, India [118].

#### 3.6.2. Health Effects

Large amounts of naphthalene in the air can irritate the eyes and respiratory system. The WHO (2010) established an annual average IAQ guideline (10 μg/m^3^) based on respiratory tract lesions, including tumors in the upper respiratory tract demonstrated in animal studies, and hemolytic anemia in humans [1]. Overall, naphthalene presents health risks in a subset of homes where inappropriate use of repellents and deodorants takes place [116].

### 3.7. Other Indoor Pollutants

A number of other indoor pollutants were examined, often in multipollutant monitoring studies. Thirteen studies measured CO, a product of incomplete combustion, in domestic indoor environments with generally low (below 1 mg/m^3^) non-acute concentrations observed in houses in USA [7,119] and England [101], but with much higher levels (mean 6 mg/m^3^) in houses close to heavy traffic in summer in Delhi, India [39], and in rooms with hookah smoking in Dubai, UAE [28]. Relatively high CO concentrations (max 8 h mean 2.5 mg/m^3^) were recorded in kitchens with gas cookers in California, USA [60].

Five studies examined O_3_ concentrations in indoor air, and those that did (e.g., in Southern California, USA [120], Canada [121], and Japan [55]) generally found very low mean indoor O_3_ levels, ranging between 0.03 and 29.7 μg/m^3^, compared with outdoor concentrations (Table S1). Ozone is a respiratory irritant formed in the atmosphere from the chemical reaction of precursor gases (NO_x_, CO, and VOCs) in the presence of sunlight. Penetrating from outdoors, O_3_ can be decomposed quickly by chemical compounds present in indoor air. Uchiyama et al. (2015) suggested that the indoor/outdoor (I/O) ratio of O_3_ can represent the ventilation of the indoor environment [55].

From the five studies which included indoor SO_2_ monitoring, detectable levels (0.6 μg/m^3^) were reported from a study in Durban, South Africa [122], with much higher levels reported from Agra (26.2–44.5 μg/m^3^) and particularly Delhi (67–220 μg/m^3^) in India [39,40] (Table S1). Concentrations of SO_2_, which is a product of combustion of sulphur-containing fuels such as diesel, kerosene and coal, were much higher during the rainy season in Delhi, particularly outdoors, compared to other seasons [39].

## 4. Key Determinants of Indoor Air Pollution

### 4.1. Housing Characteristics

Indoor air pollution levels were influenced by housing characteristics, such as type of cooking and heating system, presence and type of windows, building materials and age, and presence of attached garages (e.g., [84,88,98,123,124]). For example, housing characteristics and occupants’ activities could explain up to approximately 50% of the variability in indoor air pollutant concentrations in Canadian houses, with ventilation, home age, and attached garage being important predictors for many pollutants [121].

Ventilation in particular, often expressed as AER, was a key determinant of IAQ. AER was an important determinant of indoor formaldehyde, acetaldehyde, and acrolein levels [102], and of both indoor and personal PM_2.5_ [125]. When AERs were higher (>1 exchange/hour), the impact of indoor sources was less pronounced, as indoor PM concentrations tracked outdoor levels more closely [126]. Lower indoor PM_2.5_ levels were associated with the presence of airtight windows in winter in Italy [123] and with larger homes in California, USA [127]. Lajoie et al. (2015) suggested that enhanced ventilation improves IAQ and may prevent wheezing in children with asthma [27].

Indoor NO_2_ and PM_2.5_ levels in Italy were positively related to the presence of a gas boiler at home in summer [123]. A study in New Zealand showed that replacing unflued gas heaters was associated with a 67% reduction in NO_2_ levels in living rooms [128]. Replacing unvented gas stoves with electric stoves or placement of air purifiers with high-efficiency particulate air (HEPA) and carbon filters decreased indoor NO_2_ concentrations in homes in Baltimore, USA [129]. However, NO_2_ concentrations in the kitchen and bedroom did not significantly change following range hood installation.

Attached garages affected indoor levels of VOCs such as benzene [91]. Concentrations measured in houses with integral garages in the UK showed higher concentrations for almost all VOCs, but not for PAHs [84]. Wang et al. (2017) identified the most abundant VOCs in 25 homes in the UK, which included benzene, toluene, xylenes, d-limonene and a-pinene [90]. Although concentrations within homes varied considerably, no statistically significant association with the building age, size, single/double glazed windows, or occupancy patterns was found in this study (possibly due to the small number of houses examined).

Green eco-friendly housing, which includes approaches to reduce indoor air pollutant sources and to increase energy efficiency, reduced the penetration of outdoor pollutants. Colton et al. (2014) observed lower PM_2.5_ and NO_2_ levels in green vs. conventional apartments, and improvements in self-reported health and reductions in SBS symptoms in Boston, USA [130]. However, making houses more airtight could result in increased build up of indoor pollutants and building overheating [131,132].

Informal building structures made of low quality building materials were characterised by unregulated AERs, which were likely to contribute to indoor air pollution. In informal houses, surfaces (e.g., walls made of corrugated sheet, mud or wood) were likely to release or re-suspend particles into the air when disturbed. Informally constructed houses in a study in Durban, South Africa had higher PM_10_ concentrations when compared to formally constructed houses [122]. Gurley et al. (2013) found that each additional external window and/or door was associated with a 22 μg/m^3^ decrease on average in indoor PM_2.5_ in households in Dhaka, Bangladesh [36]. PM_10_ concentrations were significantly lower in houses in which cooking occurred on a stove with a chimney, compared to houses in which cooking occurred on stoves without chimneys in Lao PDR [38].

### 4.2. Occupancy, Natural Ventilation and Activity Patterns

Indoor pollution levels were positively associated with household and personal activities, such as cooking, smoking, use of a fireplace, stove, candles, incense or vacuum cleaners, and interior rebuilding or renovation (e.g., [132,133,134,135,136]), as well as with the number of occupants in the household [137,138]. Extremely high indoor concentrations of chemicals (naphthalene, p-dichlorobenzene) were associated with inappropriate use of insecticides and space deodorisers [55,116].

Natural ventilation and window opening were beneficial or in cases detrimental for IAQ, depending on the location of the household, the emission source and the season. Open windows were generally beneficial in houses with smokers [20,139]. In Denmark, opening of windows in infants’ bedrooms during the summer decreased PM_2.5_ concentrations, whereas open windows during winter resulted in a higher indoor concentrations possibly due to greater contributions of outdoor PM_2.5_ [139]. Open windows were associated with significantly lower indoor PM_2.5_ and PM_10_ levels in Baltimore, USA [134]. In the same study, the use of air conditioning did not have a significant impact on indoor PM_2.5_ or PM_10_ [134]. Klepeis et al. (2017) did not observe an association between particle counts and window opening, use of kitchen exhaust fans, or other ventilation activities in low-income households in San Diego, USA [127]. In a study in Italy, indoor NO_2_ concentrations increased with increasing number of hours of open windows in summer [123].

PM_2.5_ concentrations were reduced by use of a range hood for frying, by not using candles, a fireplace or a stove, by increasing the distance between the bedroom and the smoking area and by opening windows in houses of smokers in Denmark [139]. Agricultural and housing variables were poorly associated with indoor and outdoor PM_10_ and PM_2.5_ in rural Iowa, USA, except for home cleanliness which was highly associated with indoor PM_10_ [140]. Major determinants of indoor NO_2_ concentrations in two cohorts in Spain and one in the UK were the heating/cooking fuel used in the house (gas fire and gas cooker increased average NO_2_ concentrations by 1.27 and 2.13 fold, respectively), parental cigarette smoking, and season of measurement [141]. NO_x_, NO_2_ and CO were higher in homes that cooked with gas and increased with amount of gas cooking in California, USA [60].

Tobacco smoking and burning of incense were significant contributors to indoor air pollution in studies in South Africa [122] and India [142]. Vanker et al. (2015) found a significant association between the use of fossil fuels for cooking and increased benzene, CO and NO_2_ levels in Cape Town, South Africa [143]. A study by Lawrence et al. (2005) in Agra, India, suggested that indoor sources (e.g., wood burning, smoking) mainly affected indoor CO levels during winter [144]. At urban sites where NO and NO_2_ concentrations were very high, IAQ was mainly affected by outdoor sources. At these sites, the usage of heavy diesel generators and traffic pollution were the major outdoor sources affecting IAQ.

### 4.3. Seasonal and Climatic Influences

Significant seasonal patterns of indoor pollutant concentrations have been observed in homes (e.g., [55,123,145]), mainly depending on the relative strength of indoor and outdoor sources, ventilation rates, and ambient or room temperature. Temperature affected the emission rates of pollutants (e.g., VOCs) from indoor materials [55,100], as well as occupancy patterns such as opening of windows and use of heating and cooling systems. For example, indoor concentrations for most pollutants were higher in summer than in winter in a study in Japan [55]. In particular, formaldehyde, toluene, and ammonia were strongly and positively associated temperature. On the other hand, indoor benzene, SO_2_ and NO_2_ concentrations were higher in winter due to the presence of unvented gas heaters and reduced ventilation. In a study in Cairo, Egypt [146], air temperature, relative humidity and the age of the flat were the main factors affecting indoor formaldehyde levels, with higher concentrations recorded in summer.

Wind dispersed outdoor pollutants and affected AERs [147]. A study in Athens, Greece, showed that seasonal variation of wind speed was an important factor affecting toluene and xylene concentrations in homes [77]. In Italy, indoor NO_2_ and PM_2.5_ concentrations in urban and rural households were higher in winter compared to summer [123].

Significant seasonal variations of all PM fractions were observed in Delhi and Agra, India [39,142]. Particulate I/O ratios and concentrations were also linked with meteorological conditions and indoor activities. Concentration of all PM fractions were higher in winter due to increased indoor space heating and reduced pollutant dispersion in comparison with other seasons. On the other hand, indoor concentrations of CO, SO_2_ and NO_x_ in Delhi were relatively higher in the rainy or summer seasons due to the increased penetration of outdoor pollution.

Indoor PM_2.5_ concentrations in urban households in Dhaka, Bangladesh, were associated with a very large (225 μg/m^3^) increase in winter [36]. A significant seasonal association was also found with higher indoor air pollution levels in winter in Cape Town, South Africa [143]. Overall, indoor pollution sources typically have a greater impact on personal exposures during winter, when homes have reduced ventilation and residents spend more time indoors [148].

### 4.4. Dwelling Location, Setting and Within-Building Variability

The building location within a city (e.g., distance from major road) and setting (urban, suburban or rural) had a strong influence on IAQ, particularly in relation to air pollutants of outdoor origin. PM_2.5_ and PM_10_ levels were nearly twice as high in urban compared to rural homes in Malaysia [149], and significantly higher in suburban compared to downtown homes in Slovakia [150], Italy [123] and USA [151]. Roadside dwellings in Chinese cities had higher indoor PM_2.5_ and elemental carbon (EC) levels compared to other urban dwellings, indicating the influence of outdoor traffic emissions [32,33]. PM_2.5_ levels in six Chinese cities showed strong correlation between living room, bedroom, and outdoor PM_2.5_, suggesting that indoor concentrations were strongly influenced by outdoor sources [32]. Another study from northwest China found a significant positive correlation between kitchen and bedroom PM_2.5_, suggesting that they were affected by similar pollution sources [34].

Location also played a role in indoor NO_2_ and VOC levels in homes in Europe and USA [151,152,153]. Urban dwellings in France were found to be more polluted than rural ones, with concentrations approximately 2-fold higher for NO_2_, benzene, toluene, ethylbenzene in urban homes [152]. Indoor benzene, toluene, and xylene levels were influenced by location (i.e., centre or suburb), proximity to busy road and proximity to petrol station in Athens, Greece, with proximity to petrol station or busy road having the greatest impact on indoor levels of benzene possibly due to higher evaporative emissions [77,153]. However, in a UK study, homes located on roadsides and homes located away from traffic had similar concentrations for high-molecular-weight PAHs and almost all VOCs, except toluene which was significantly higher in first-line homes [84].

There was limited evidence of IAQ variability within the same residence or building [30,45,62,104,145]. For example, significant positive correlations between formaldehyde in bedrooms and living rooms were reported for homes in Spain [104], between PM_2.5_ in kitchens and living rooms in the UK [30], and between NO_2_ in bedrooms and living rooms in USA [62]. In a study in Western Australia, Jones et al. [154] showed that there were no significant differences between levels of PM measured at two different heights in the living room, and between living room and bedroom levels. In a study in Lao PDR, Morawska et al. [38] found no significant differences in PM_10_ levels as a function of cooking location within homes. However, Nasir and Colbeck [155] found higher PM levels in UK houses with open plan kitchens compared to those with separate kitchens. In Boston, USA [156,157], there was transfer of second-hand smoke within multiunit residential buildings, which had an impact on PM_2.5_ levels in smoke-free apartments directly adjacent to smoking households.

## 5. Discussion

This review focused on indoor air pollution levels, influencing factors and related health effects in domestic dwellings. We systematically reviewed the global scientific literature and extracted data from 141 eligible studies published over eighteen years. Concentrations of most indoor pollutants varied very widely, in cases exceeding WHO air quality guidelines (Table 1) [1,158,159]. The reviewed evidence suggests that even in highly polluted urban environments, indoor activities play a major role in indoor exposure, particularly cigarette smoking for PM_2.5_, unvented gas appliances for NO_2_, moth repellents for naphthalene, and household products and materials for VOCs including formaldehyde.

The I/O concentration ratios (e.g., of PM_2.5_, BTEX) typically exceeded 1 in households with smokers. Formaldehyde was typically present at higher concentration indoors than outdoors, with indoor levels positively correlated with temperature and negatively correlated with age of building. In most studies, AERs were negatively associated with indoor air pollution levels. Built environment characteristics, such as homes near high-traffic-density roads, redecoration, small house size, and informal building structure contributed to high indoor air pollution levels.

Indoor NO_2_ and PM_2.5_ exposures were associated with the presence of acute respiratory symptoms and mild lung function impairment, respectively. Exposure to indoor NO_2_ is of particular concern in relation to the respiratory health of children with asthma. Interventions aimed at lowering indoor NO_2_ concentrations, particularly in inner-city homes, may reduce asthma morbidity in children. Reducing the use of unvented gas heating in homes would substantially lower indoor NO_2_ exposure.

Improved stoves with chimneys, use of cleaner household fuels, and better housing and kitchen design with improved ventilation will help control indoor air pollution. Behavioural interventions, such as raising awareness for reducing smoking and ETS exposure, reducing emissions from stoves and modified cooking practices (e.g., use of range hoods) will help in reducing indoor pollution levels. A large proportion of indoor carbonaceous PM (particularly EC) comes from outdoor sources in highly polluted urban environments. Therefore, it is critical to control outdoor emissions from motor vehicles to improve IAQ [160]. In highly polluted roadside environments, natural ventilation strategies should take into account the location of windows and time of opening to reduce ingress of outdoor pollutants.

Interventions to reduce biomass burning for cooking and heating could result in a substantial reduction in indoor PM_2.5_ levels in lower income urban households. In high-income settings, there is a trend towards more airtight and smaller dwellings, often with an open plan configuration, which in the presence of indoor pollution sources may result in higher exposures to indoor air pollutants. Domestic exposure to PM, NO_2_ and VOCs at levels even below current recommendations may increase the risk of childhood asthma. Therefore, it is important to better control indoor emissions, including PM_2.5_ from smoking, NO_2_ from gas appliances, formaldehyde from building materials and furnishings, and BTEX from integrated garages.

Low-income dwellings are often disproportionately affected by indoor air pollution due to inadequate ventilation, overcrowding, and greater contributions from indoor (e.g., smoking) and outdoor (e.g., heavy road traffic) pollution sources [137,161,162]. Therefore, isolated interventions may not be sufficient to reduce interrelated exposures to air pollutants from multiple sources, particularly in low-income dwellings. Holistic system-based approaches are required to reduce health burdens and inequalities associated with exposure to chemical air pollutants, microbial contamination and overheating in residences, as well as reduce the carbon footprint of the housing sector [130,131,163,164]. These approaches involve controlling indoor sources of chemicals, improving ventilation, and providing air filtration [101,165,166,167].

As pointed out by other authors [9,168], we still know much less about IAQ than we do about outdoor air quality. More comprehensive and standardised IAQ data and measurement methods [169], and information on related health effects, including synergistic effects of multipollutant exposures, are needed, particularly from countries undergoing rapid economic and demographic change in Africa, Central Asia, and Latin America.

## 6. Conclusions

Although most people spend the largest proportion of their time indoors, information about levels of IAQ, particularly in homes, is sparse and less accessible than for outdoor air quality. We systematically reviewed 141 IAQ studies in homes in 29 countries published over a period of 18 years (2000–2017). Indoor levels of most air pollutants were very variable and largely depended on the strength of indoor sources, such as tobacco smoking, unvented gas appliances and certain household products (e.g., moth repellents), proximity of residence to road traffic, and room ventilation rates. Intervention to improve IAQ, for example by removing indoor pollution sources, providing adequate mechanical ventilation with air filtration or increasing natural ventilation during periods of the day when outdoor air pollution is low, are likely to benefit occupants’ respiratory health and reduce asthma morbidity in children. Standardized IAQ measurement and analytical methods, and longer monitoring periods over multiple sites are needed to develop a more comprehensive understanding of the complex factors that influence air quality in the home environment.

## Figures and Tables

**Table 1:** Mean indoor air pollutant concentrations (μg/m^3^) reported in the reviewed studies and relevant indoor and/or outdoor World Health Organization guidelines [1,158,159].

Pollutant	Range (indoor)	WHO (indoor)	WHO (outdoor)
PM_2.5_	1.7–428.6	-	25/10 ^e^
PM_10_	11.0–1275	-	50/20 ^e^
NO_2_	3.4–1210	200/40 ^a^	200/40 ^a^
Benzene	0.6–24.8	1.7 ^b^	1.7 ^b^
Toluene	3.0–325.5	-	1000/260 ^f^
Formaldehyde	7.5–134.0	100 ^c^	100 ^c^
Naphthalene	0.12–26.3	10 ^d^	-

^a^ 1 h average/annual mean guidelines recommended by the WHO (2005; 2010) for outdoor and indoor air quality. ^b^ Concentration associated with an excess lifetime risk of 1/100,000 in the WHO (2000; 2010) guidelines for outdoor and indoor air quality. ^c^ Not to be exceeded at any 30 min interval during a day recommended by the WHO (2005; 2010) for outdoor and indoor air quality. ^d^ Annual mean guideline recommended by the WHO (2010) for indoor air quality. ^e^ The 24 h/annual mean guidelines recommended by the WHO (2005) for outdoor air quality. ^f^ 30 min/7 day average guidelines recommended by the WHO (2000) for outdoor air quality.

## Supplementary Materials

The following are available online at https://www.mdpi.com/1660-4601/17/23/8972/s1, Search strategy; Search terms; Table S1: Summary of eligible study findings. References [2,7,16,17,18,19,20,21,22,23,24,25,26,27,28,29,30,31,32,33,34,35,36,37,38,39,40,41,42,43,44,45,46,47,48,49,50,51,52,53,54,55,56,57,58,59,60,61,62,63,64,65,66,67,68,69,70,71,72,73,74,75,76,77,78,79,80,81,82,83,84,85,86,87,88,89,90,91,92,93,94,95,96,97,98,99,100,101,102,103,104,105,107,108,110,111,112,113,114,116,118,119,120,121,122,123,124,125,126,127,128,129,130,132,133,134,135,136,137,138,139,140,141,142,143,144,145,146,147,148,149,150,151,152,153,154,155,156,157,159,160,169] are cited in the supplementary materials.

## References

[1] World Health Organization (2010). WHO Guidelines for Indoor Air Quality: Selected Pollutants.

[2] Lai H.K., Kendall M., Ferrier H., Lindup I., Alm S., Hänninen O., Jantunen M., Mathys P., Colvile R., Ashmore M.R. (2004). Personal exposures and microenvironment concentrations of PM_2.5_, VOC, NO_2_ and CO in Oxford, UK. Atmos. Environ..

[3] Vardoulakis S., Kinney P. (2019). Grand Challenges in Sustainable Cities and Health. Front. Sustain. Cities.

[4] de Juniac A., Kreis I., Ibison J., Murray V. (2012). Epidemiology of unintentional carbon monoxide fatalities in the UK. Int. J. Environ. Health Res..

[5] Sarigiannis D.A., Karakitsios S.P., Gotti A., Liakos I.L., Katsoyiannis A. (2011). Exposure to major volatile organic compounds and carbonyls in European indoor environments and associated health risk. Environ. Int..

[6] Jordan R.E., Cheng K.K., Miller M.R., Adab P. (2011). Passive smoking and chronic obstructive pulmonary disease: Cross-sectional analysis of data from the Health Survey for England. BMJ Open.

[7] Blanc P.D., Eisner M.D., Katz P.P., Yen I.H., Archea C., Earnest G., Janson S., Masharani U.B., Quinlan P.J., Hammond S.K. (2005). Impact of the home indoor environment on adult asthma and rhinitis. J. Occup. Environ. Med..

[8] Rushton L. (2004). Health impact of environmental tobacco smoke in the home. Rev. Environ. Health.

[9] Jones A.P. (1999). Indoor air quality and health. Atmos. Environ..

[10] Vardoulakis S., Fisher B.E.A., Pericleous K., Gonzalez-Flesca N. (2003). Modelling air quality in street canyons: A review. Atmos. Environ..

[11] Crump D., Brown V., Rowley J., Squire R. (2004). Reducing ingress of organic vapours into homes situated on contaminated land. Environ. Technol..

[12] Vardoulakis S., Dimitrova R., Richards K., Hamlyn D., Camilleri G., Weeks M., Sini J.-F., Britter R., Borrego C., Schatzmann M. (2011). Numerical Model Inter-comparison for Wind Flow and Turbulence Around Single-Block Buildings. Environ. Model. Assess..

[13] Hall D.J.S., Spanton A.M. (2012). Ingress of External Contaminants into Buildings–A Review. Atmospheric Dispersion Modelling Liaison Committee. https://admlc.files.wordpress.com/2014/05/admlc-r7-2012-1.pdf.

[14] Milner J., Vardoulakis S., Chalabi Z., Wilkinson P. (2011). Modelling inhalation exposure to combustion-related air pollutants in residential buildings: Application to health impact assessment. Environ. Int..

[15] Nazaroff W. (2013). Exploring the consequences of climate change for indoor air quality*. Environ. Res. Lett..

[16] Ryan P.H., Brokamp C., Fan Z.H., Rao M.B. (2015). Analysis of personal and home characteristics associated with the elemental composition of PM_2.5_ in indoor, outdoor, and personal air in the RIOPA Study. Res. Rep. Health Eff. Inst..

[17] Clougherty J.E., Houseman E.A., Levy J.I. (2011). Source apportionment of indoor residential fine particulate matter using land use regression and constrained factor analysis. Indoor Air.

[18] MacNeill M., Kearney J., Wallace L., Gibson M., Héroux M.E., Kuchta J., Guernsey J.R., Wheeler A.J. (2014). Quantifying the contribution of ambient and indoor-generated fine particles to indoor air in residential environments. Indoor Air.

[19] Mohammadyan M., Ashmore M. (2005). Personal Exposure and Indoor PM_2.5_ Concentrations in an Urban Population. Indoor Built Environ..

[20] Wallace L., Mitchell H., O’Connor G., Neas L., Lippmann M., Kattan M., Koenig J., Stout J., Vaughn B., Wallace D. (2003). Particle concentrations in inner-city homes of children with asthma: The effect of smoking, cooking, and outdoor pollution. Environ. Health Perspect..

[21] Rojas-Bracho L., Suh H.H., Catalano P.J., Koutrakis P. (2004). Personal exposures to particles and their relationships with personal activities for chronic obstructive pulmonary disease patients living in Boston. J. Air Waste Manag. Assoc..

[22] Rosen L., Zucker D., Hovell M., Brown N., Ram A., Myers V. (2015). Feasibility of Measuring Tobacco Smoke Air Pollution in Homes: Report from a Pilot Study. Int. J. Environ. Res. Public Health.

[23] Wyss A.B., Jones A.C., Bølling A.K., Kissling G.E., Chartier R., Dahlman H.J., Rodes C.E., Archer J., Thornburg J., Schwarze P.E. (2016). PM_2.5_ exposure and self-reported use of wood stoves and other indoor combustion sources in urban nonsmoking homes in Norway. PLoS ONE.

[24] Brown D.R., Alderman N., Weinberger B., Lewis C., Bradley J., Curtis L. (2014). Outdoor wood furnaces create significant indoor particulate pollution in neighboring homes. Inhal. Toxicol..

[25] McNamara M., Thornburg J., Semmens E., Ward T., Noonan C. (2013). Coarse particulate matter and airborne endotoxin within wood stove homes. Indoor Air.

[26] Mills L.M., Semple S.E., Wilson I.S., MacCalman L., Amos A., Ritchie D., O’Donnell R., Shaw A., Turner S.W. (2012). Factors influencing exposure to secondhand smoke in preschool children living with smoking mothers. Nicotine Tob. Res..

[27] Lajoie P., Aubin D., Gingras V., Daigneault P., Ducharme F., Gauvin D., Fugler D., Leclerc J.M., Won D., Courteau M. (2015). The IVAIRE project—A randomized controlled study of the impact of ventilation on indoor air quality and the respiratory symptoms of asthmatic children in single family homes. Indoor Air.

[28] Weitzman M., Yusufali A.H., Bali F., Vilcassim M.J.R., Gandhi S., Peltier R., Nadas A., Sherman S., Lee L., Hong Z. (2016). Effects of hookah smoking on indoor air quality in homes. Tob. Control.

[29] Jones N.C., Thornton C.A., Mark D., Harrison R.M. (2000). Indoor/outdoor relationships of particulate matter in domestic homes with roadside, urban and rural locations. Atmos. Environ..

[30] Wigzell E., Kendall M., Nieuwenhuijsen M.J. (2000). The spatial and temporal variation of particulate matter within the home. J. Expo. Sci. Environ. Epidemiol..

[31] Tunno B.J., Kyra Naumoff S., Cambal L., Tripathy S., Holguin F., Lioy P., Clougherty J.E. (2015). Indoor air sampling for fine particulate matter and black carbon in industrial communities in Pittsburgh. Sci. Total Environ..

[32] Hu J., Li N., Lv Y., Liu J., Xie J., Zhang H. (2017). Investigation on Indoor Air Pollution and Childhood Allergies in Households in Six Chinese Cities by Subjective Survey and Field Measurements. Int. J. Environ. Res. Public Health.

[33] Lai S., Ho K.F., Zhang Y., Lee S., Huang Y., Zou S. (2010). Characteristics of Residential Indoor Carbonaceous Aerosols: A Case Study in Guangzhou, Pearl River Delta Region. Aerosol Air Qual. Res..

[34] Li T., Cao S., Fan D., Zhang Y., Wang B., Zhao X., Leaderer B.P., Shen G., Zhang Y., Duan X. (2016). Household concentrations and personal exposure of PM_2.5_ among urban residents using different cooking fuels. Sci. Total Environ..

[35] Cao J.J., Lee S.C., Chow J.C., Cheng Y., Ho K.F., Fung K., Liu S.X., Watson J.G. (2005). Indoor/outdoor relationships for PM_2.5_ and associated carbonaceous pollutants at residential homes in Hong Kong-case study. Indoor Air.

[36] Gurley E.S., Salje H., Homaira N., Ram P.K., Haque R., Petri W.A., Bresee J., Moss W.J., Luby S.P., Breysse P. (2013). Seasonal concentrations and determinants of indoor particulate matter in a low-income community in Dhaka, Bangladesh. Environ. Res.

[37] Lévesque B., Allaire S., Gauvin D., Koutrakis P., Gingras S., Rhainds M., Prud’Homme H., Duchesne J.-F. (2001). Wood-burning appliances and indoor air quality. Sci. Total Environ..

[38] Morawska L., Mengersen K., Wang H., Tayphasavanh F., Darasavong K., Holmes N.S. (2011). Pollutant concentrations within households in Lao PDR and association with housing characteristics and occupants’ activities. Environ. Sci. Technol..

[39] Kulshreshtha P., Khare M., Seetharaman P. (2008). Indoor air quality assessment in and around urban slums of Delhi city, India. Indoor Air.

[40] Taneja A., Saini R., Masih A. (2008). Indoor air quality of houses located in the urban environment of Agra, India. Ann. N. Y. Acad. Sci..

[41] Custódio D., Pinho I., Cerqueira M., Nunes T., Pio C. (2014). Indoor and outdoor suspended particulate matter and associated carbonaceous species at residential homes in northwestern Portugal. Sci. Total Environ..

[42] BéruBé K.A., Sexton K.J., Jones T.P., Moreno T., Anderson S., Richards R.J. (2004). The spatial and temporal variations in PM_10_ mass from six UK homes. Sci. Total Environ..

[43] Chen Y., Li X., Zhu T., Han Y., Lv D. (2017). PM_2.5_-bound PAHs in three indoor and one outdoor air in Beijing: Concentration, source and health risk assessment. Sci. Total Environ..

[44] Wallace L. (2006). Indoor sources of ultrafine and accumulation mode particles: Size distributions, size-resolved concentrations, and source strengths. Aerosol Sci. Technol..

[45] Karottki D.G., Spilak M., Frederiksen M., Jovanovic Andersen Z., Madsen A.M., Ketzel M., Massling A., Gunnarsen L., Møller P., Loft S. (2015). Indoor and outdoor exposure to ultrafine, fine and microbiologically derived particulate matter related to cardiovascular and respiratory effects in a panel of elderly urban citizens. Int. J. Environ. Res. Public Health.

[46] Bhangar S., Mullen N.A., Hering S.V., Kreisberg N.M., Nazaroff W.W. (2011). Ultrafine particle concentrations and exposures in seven residences in northern California. Indoor Air.

[47] Weichenthal S., Dufresne A., Infante-Rivard C., Joseph L. (2007). Indoor ultrafine particle exposures and home heating systems: A cross-sectional survey of Canadian homes during the winter months. J. Expo. Sci. Environ. Epidemiol..

[48] Olsen Y., Karottki D.G., Jensen D.M., Bekö G., Kjeldsen B.U., Clausen G., Hersoug L.-G., Holst G.J., Wierzbicka A., Sigsgaard T. (2014). Vascular and lung function related to ultrafine and fine particles exposure assessed by personal and indoor monitoring: A cross-sectional study. Environ. Health.

[49] McCormack M.C., Breysse P.N., Matsui E.C., Hansel N.N., Williams D., Curtin-Brosnan J., Eggleston P., Diette G.B. (2009). In-home particle concentrations and childhood asthma morbidity. Environ. Health Perspect..

[50] McCormack M.C., Breysse P.N., Matsui E.C., Hansel N.N., Peng R.D., Curtin-Brosnan J., Williams D.L., Wills-Karp M., Diette G.B. (2011). Indoor particulate matter increases asthma morbidity in children with non-atopic and atopic asthma. Ann. Allergy Asthma Immunol..

[51] Jung K.H., Hsu S.I., Yan B., Moors K., Chillrud S.N., Ross J., Wang S., Perzanowski M.S., Kinney P.L., Whyatt R.M. (2012). Childhood exposure to fine particulate matter and black carbon and the development of new wheeze between ages 5 and 7 in an urban prospective cohort. Environ. Int..

[52] Delfino R., Quintana P., Floro J., Gastañaga V., Samimi B., Kleinman M., Liu L.J., Bufalino C., Wu C.-F., McLaren C. (2004). Association of FEV1 in Asthmatic Children with Personal and Microenvironmental Exposure to Airborne Particulate Matter. Environ. Health Perspect..

[53] Chi M.C., Guo S.E., Hwang S.L., Chou C.T., Lin C.M., Lin Y.C. (2016). Exposure to Indoor Particulate Matter Worsens the Symptoms and Acute Exacerbations in Chronic Obstructive Pulmonary Disease Patients of Southwestern Taiwan: A Pilot Study. Int. J. Environ. Res. Public Health.

[54] Osman L.M., Douglas J.G., Garden C., Reglitz K., Lyon J., Gordon S., Ayres J.G. (2007). Indoor air quality in homes of patients with chronic obstructive pulmonary disease. Am. J. Respir. Crit. Care Med..

[55] Uchiyama S., Tomizawa T., Tokoro A., Aoki M., Hishiki M., Yamada T., Tanaka R., Sakamoto H., Yoshida T., Bekki K. (2015). Gaseous chemical compounds in indoor and outdoor air of 602 houses throughout Japan in winter and summer. Environ. Res..

[56] Hansel N., McCormack M., Belli A., Matsui E., Peng R., Aloe C., Paulin L., Williams D.A., Diette G., Breysse P. (2013). In-home air pollution is linked to respiratory morbidity in former smokers with COPD. Am. J. Respir. Crit. Care Med..

[57] Belanger K., Holford T.R., Gent J.F., Hill M.E., Kezik J.M., Leaderer B.P. (2013). Household levels of nitrogen dioxide and pediatric asthma severity. Epidemiology.

[58] Kornartit C., Sokhi R.S., Burton M.A., Ravindra K. (2010). Activity pattern and personal exposure to nitrogen dioxide in indoor and outdoor microenvironments. Environ. Int..

[59] Cyrys J., Heinrich J., Richter K., Wölke G., Wichmann H.E. (2000). Sources and concentrations of indoor nitrogen dioxide in Hamburg (west Germany) and Erfurt (east Germany). Sci. Total Environ..

[60] Mullen N.A., Li J., Russell M.L., Spears M., Less B.D., Singer B.C. (2016). Results of the California Healthy Homes Indoor Air Quality Study of 2011–2013: Impact of natural gas appliances on air pollutant concentrations. Indoor Air.

[61] Cibella F., Cuttitta G., Della Maggiore R., Ruggieri S., Panunzi S., De Gaetano A., Bucchieri S., Drago G., Melis M.R., La Grutta S. (2015). Effect of indoor nitrogen dioxide on lung function in urban environment. Environ. Res..

[62] Zipprich J.L., Harris S.A., Fox J.C., Borzelleca J.F. (2002). An analysis of factors that influence personal exposure to nitrogen oxides in residents of Richmond, Virginia. J. Expo. Anal. Environ. Epidemiol..

[63] Belanger K., Gent J.F., Triche E.W., Bracken M.B., Leaderer B.P. (2006). Association of indoor nitrogen dioxide exposure with respiratory symptoms in children with asthma. Am. J. Respir. Crit. Care Med..

[64] Hansel N., Breysse P., McCormack M., Matsui E., Curtin-Brosnan J., Williams D.A., Moss J., Cuhran J., Diette G. (2008). A Longitudinal Study of Indoor Nitrogen Dioxide Levels and Respiratory Symptoms in Inner-City Children with Asthma. Environ. Health Perspect..

[65] Kattan M., Gergen P., Eggleston P., Visness C., Mitchell H. (2007). Health effects of indoor nitrogen dioxide and passive smoking on urban asthmatic children. J. Allergy Clin. Immunol..

[66] Gillespie-Bennett J., Pierse N., Wickens K., Crane J., Howden-Chapman P. (2011). The respiratory health effects of nitrogen dioxide in children with asthma. Eur. Respir. J..

[67] Paulin L.M., Williams D.L., Peng R., Diette G.B., McCormack M.C., Breysse P., Hansel N.N. (2017). 24-h Nitrogen dioxide concentration is associated with cooking behaviors and an increase in rescue medication use in children with asthma. Environ. Res..

[68] Delgado-Saborit J.M., Aquilina N.J., Meddings C., Baker S., Harrison R.M. (2011). Relationship of personal exposure to volatile organic compounds to home, work and fixed site outdoor concentrations. Sci. Total Environ..

[69] Tanaka-Kagawa T., Uchiyama S., Matsushima E., Sasaki A., Kobayashi H., Kobayashi H., Yagi M., Tsuno M., Arao M., Ikemoto K. (2005). Survey of volatile organic compounds found in indoor and outdoor air samples from Japan. Bull. Natl. Inst. Health Sci..

[70] Wheeler A.J., Wong S.L., Khouri C., Zhu J. (2013). Predictors of indoor BTEX concentrations in Canadian residences. Health Rep..

[71] Cheng M., Galbally I.E., Molloy S.B., Selleck P.W., Keywood M.D., Lawson S.J., Powell J.C., Gillett R.W., Dunne E. (2016). Factors controlling volatile organic compounds in dwellings in Melbourne, Australia. Indoor Air.

[72] Adgate J.L., Eberly L.E., Stroebel C., Pellizzari E.D., Sexton K. (2004). Personal, indoor, and outdoor VOC exposures in a probability sample of children. J. Expo. Anal. Environ. Epidemiol..

[73] Chin J.Y., Godwin C., Parker E., Robins T., Lewis T., Harbin P., Batterman S. (2014). Levels and sources of volatile organic compounds in homes of children with asthma. Indoor Air.

[74] Rumchev K., Spickett J., Bulsara M., Phillips M., Stick S. (2004). Association of domestic exposure to volatile organic compounds with asthma in young children. Thorax.

[75] Kliucininkas L., Martuzevicius D., Krugly E., Prasauskas T., Kauneliene V., Molnar P., Strandberg B. (2011). Indoor and outdoor concentrations of fine particles, particle-bound PAHs and volatile organic compounds in Kaunas, Lithuania. J. Environ. Monit..

[76] Villanueva F., Tapia A., Amo-Salas M., Notario A., Cabañas B., Martínez E. (2015). Levels and sources of volatile organic compounds including carbonyls in indoor air of homes of Puertollano, the most industrialized city in central Iberian Peninsula. Estimation of health risk. Int. J. Hyg. Environ. Health.

[77] Alexopoulos E.C., Chatzis C., Linos A. (2006). An analysis of factors that influence personal exposure to toluene and xylene in residents of Athens, Greece. BMC Public Health.

[78] Lee J.H., Lee H.S., Park M.R., Lee S.W., Kim E.H., Cho J.B., Kim J., Han Y., Jung K., Cheong H.K. (2014). Relationship between indoor air pollutant levels and residential environment in children with atopic dermatitis. Allergy Asthma Immunol. Res..

[79] Ferrero A., Esplugues A., Estarlich M., Llop S., Cases A., Mantilla E., Ballester F., Iñiguez C. (2017). Infants’ indoor and outdoor residential exposure to benzene and respiratory health in a Spanish cohort. Environ. Pollut..

[80] Adgate J.L., Church T.R., Ryan A.D., Ramachandran G., Fredrickson A.L., Stock T.H., Morandi M.T., Sexton K. (2004). Outdoor, indoor, and personal exposure to VOCs in children. Environ. Health Perspect..

[81] Phillips M.L., Esmen N.A., Hall T.A., Lynch R. (2005). Determinants of exposure to volatile organic compounds in four Oklahoma cities. J. Expo. Anal. Environ. Epidemiol..

[82] Du L., Batterman S., Godwin C., Rowe Z., Chin J.Y. (2015). Air exchange rates and migration of VOCs in basements and residences. Indoor Air.

[83] Saijo Y., Kishi R., Sata F., Katakura Y., Urashima Y., Hatakeyama A., Kobayashi S., Jin K., Kurahashi N., Kondo T. (2004). Symptoms in relation to chemicals and dampness in newly built dwellings. Int. Arch. Occup. Environ. Health.

[84] Harrison R.M., Delgado-Saborit J.M., Baker S.J., Aquilina N., Meddings C., Harrad S., Matthews I., Vardoulakis S., Anderson H.R. (2009). Measurement and modeling of exposure to selected air toxics for health effects studies and verification by biomarkers. Res. Rep. Health Eff. Inst..

[85] Saraga D.E., Maggos T., Helmis C.G., Michopoulos J., Bartzis J.G., Vasilakos C. (2010). PM1 and PM_2.5_ ionic composition and VOCs measurements in two typical apartments in Athens, Greece: Investigation of smoking contribution to indoor air concentrations. Environ. Monit. Assess..

[86] Schneider P., Gebefügi I., Richter K., Wölke G., Schnelle J., Wichmann H.E., Heinrich J. (2001). Indoor and outdoor BTX levels in German cities. Sci. Total Environ..

[87] Jurvelin J.A., Edwards R.D., Vartiainen M., Pasanen P., Jantunen M.J. (2003). Residential Indoor, Outdoor, and Workplace Concentrations of Carbonyl Compounds: Relationships with Personal Exposure Concentrations and Correlation with Sources. J. Air Waste Manag. Assoc..

[88] Park J.S., Ikeda K. (2006). Variations of formaldehyde and VOC levels during 3 years in new and older homes. Indoor Air.

[89] Guo H., Kwok N.H., Cheng H.R., Lee S.C., Hung W.T., Li Y.S. (2009). Formaldehyde and volatile organic compounds in Hong Kong homes: Concentrations and impact factors. Indoor Air.

[90] Wang C.M., Barratt B., Carslaw N., Doutsi A., Dunmore R., Ward M., Lewis A. (2017). Unexpectedly high concentrations of monoterpenes in a study of UK homes. Environ. Sci. Process. Impacts.

[91] Gordian M.E., Stewart A.W., Morris S.S. (2010). Evaporative gasoline emissions and asthma symptoms. Int. J. Environ. Res. Public Health.

[92] Singleton R., Salkoski A.J., Bulkow L., Fish C., Dobson J., Albertson L., Skarada J., Kovesi T., McDonald C., Hennessy T.W. (2017). Housing characteristics and indoor air quality in households of Alaska Native children with chronic lung conditions. Indoor Air.

[93] Madureira J., Paciência I., Cavaleiro-Rufo J., Fernandes Ede O. (2016). Indoor air risk factors for schoolchildren’s health in Portuguese homes: Results from a case-control survey. J. Toxicol. Environ. Health Part A.

[94] Kwon J.H., Kim E., Chang M.H., Park E.A., Hong Y.C., Ha M., Park H., Kim Y., Park C., Ha E.H. (2015). Indoor total volatile organic compounds exposure at 6 months followed by atopic dermatitis at 3 years in children. Pediatric Allergy Immunol..

[95] Takeda M., Saijo Y., Yuasa M., Kanazawa A., Araki A., Kishi R. (2009). Relationship between sick building syndrome and indoor environmental factors in newly built Japanese dwellings. Int. Arch. Occup. Environ. Health.

[96] Marchand C., Bulliot B., Le Calvé S., Mirabel P. (2006). Aldehyde measurements in indoor environments in Strasbourg (France). Atmos. Environ..

[97] Lovreglio P., Carrus A., Iavicoli S., Drago I., Persechino B., Soleo L. (2009). Indoor formaldehyde and acetaldehyde levels in the province of Bari, South Italy, and estimated health risk. J. Environ. Monit..

[98] Maruo Y.Y., Yamada T., Nakamura J., Izumi K., Uchiyama M. (2010). Formaldehyde measurements in residential indoor air using a developed sensor element in the Kanto area of Japan. Indoor Air.

[99] Takigawa T., Saijo Y., Morimoto K., Nakayama K., Shibata E., Tanaka M., Yoshimura T., Chikara H., Kishi R. (2012). A longitudinal study of aldehydes and volatile organic compounds associated with subjective symptoms related to sick building syndrome in new dwellings in Japan. Sci. Total Environ..

[100] Clarisse B., Laurent A.M., Seta N., Le Moullec Y., El Hasnaoui A., Momas I. (2003). Indoor aldehydes: Measurement of contamination levels and identification of their determinants in Paris dwellings. Environ. Res..

[101] Raw G.J., Coward S.K., Brown V.M., Crump D.R. (2004). Exposure to air pollutants in English homes. J. Expo. Anal. Environ. Epidemiol..

[102] Gilbert N.L., Guay M., David Miller J., Judek S., Chan C.C., Dales R.E. (2005). Levels and determinants of formaldehyde, acetaldehyde, and acrolein in residential indoor air in Prince Edward Island, Canada. Environ. Res..

[103] Gilbert N.L., Gauvin D., Guay M., Héroux M.-È., Dupuis G., Legris M., Chan C.C., Dietz R.N., Lévesque B. (2006). Housing characteristics and indoor concentrations of nitrogen dioxide and formaldehyde in Quebec City, Canada. Environ. Res..

[104] Rovira J., Roig N., Nadal M., Schuhmacher M., Domingo J.L. (2016). Human health risks of formaldehyde indoor levels: An issue of concern. J. Environ. Sci. Health Part A.

[105] Rancière F., Dassonville C., Roda C., Laurent A.M., Le Moullec Y., Momas I. (2011). Contribution of ozone to airborne aldehyde formation in Paris homes. Sci. Total Environ..

[106] Herbarth O., Fritz G.J., Rehwagen M., Richter M., Röder S., Schlink U. (2006). Association between indoor renovation activities and eczema in early childhood. Int. J. Hyg. Environ. Health.

[107] Yeatts K.B., El-Sadig M., Leith D., Kalsbeek W., Al-Maskari F., Couper D., Funk W.E., Zoubeidi T., Chan R.L., Trent C.B. (2012). Indoor air pollutants and health in the United Arab Emirates. Environ. Health Perspect..

[108] Takigawa T., Wang B.L., Saijo Y., Morimoto K., Nakayama K., Tanaka M., Shibata E., Yoshimura T., Chikara H., Ogino K. (2010). Relationship between indoor chemical concentrations and subjective symptoms associated with sick building syndrome in newly built houses in Japan. Int. Arch. Occup. Environ. Health.

[109] Kume K., Ohura T., Noda T., Amagai T., Fusaya M. (2007). Seasonal and spatial trends of suspended-particle associated polycyclic aromatic hydrocarbons in urban Shizuoka, Japan. J. Hazard. Mater..

[110] Liu Y., Zhu L., Shen X. (2001). Polycyclic Aromatic Hydrocarbons (PAHs) in Indoor and Outdoor Air of Hangzhou, China. Environ. Sci. Technol..

[111] Ohura T., Sugiyama T., Amagai T., Fusaya M., Matsushita H. (2002). Simultaneous liquid chromatographic determination of 39 polycyclic aromatic hydrocarbons in indoor and outdoor air and application to a survey on indoor air pollution in Fuji, Japan. J. AOAC Int..

[112] Kennedy K., Macova M., Leusch F., Bartkow M.E., Hawker D.W., Zhao B., Denison M.S., Mueller J.F. (2009). Assessing indoor air exposures using passive sampling with bioanalytical methods for estrogenicity and aryl hydrocarbon receptor activity. Anal. Bioanal. Chem..

[113] Lu H., Amagai T., Ohura T. (2011). Comparison of polycyclic aromatic hydrocarbon pollution in Chinese and Japanese residential air. J. Environ. Sci..

[114] Cirillo T., Montuori P., Mainardi P., Russo I., Triassi M., Amodio-Cocchieri R. (2006). Multipathway Polycyclic Aromatic Hydrocarbon and Pyrene Exposure Among Children Living in Campania (Italy). J. Environ. Sci. Health Part A.

[115] Brender J.D., Pichette J.L., Suarez L., Hendricks K.A., Holt M. (2003). Health risks of residential exposure to polycyclic aromatic hydrocarbons. Arch. Environ. Health.

[116] Batterman S., Chin J.-Y., Jia C., Godwin C., Parker E., Robins T., Max P., Lewis T. (2012). Sources, concentrations and risks of naphthalene in indoor and outdoor air. Indoor Air.

[117] Jia C., Batterman S. (2010). A critical review of naphthalene sources and exposures relevant to indoor and outdoor air. Int. J. Environ. Res. Public Health.

[118] Masih J., Masih A., Kulshrestha A., Singhvi R., Taneja A. (2010). Characteristics of polycyclic aromatic hydrocarbons in indoor and outdoor atmosphere in the North central part of India. J. Hazard. Mater..

[119] Pickett A.R., Bell M.L. (2011). Assessment of indoor air pollution in homes with infants. Int. J. Environ. Res. Public Health.

[120] Lee K., Xue J., Geyh A.S., Ozkaynak H., Leaderer B.P., Weschler C.J., Spengler J.D. (2002). Nitrous acid, nitrogen dioxide, and ozone concentrations in residential environments. Environ. Health Perspect..

[121] Héroux M.E., Clark N., Van Ryswyk K., Mallick R., Gilbert N.L., Harrison I., Rispler K., Wang D., Anastassopoulos A., Guay M. (2010). Predictors of indoor air concentrations in smoking and non-smoking residences. Int. J. Environ. Res. Public Health.

[122] Jafta N., Barregard L., Jeena P.M., Naidoo R.N. (2017). Indoor air quality of low and middle income urban households in Durban, South Africa. Environ. Res..

[123] Simoni M., Scognamiglio A., Carrozzi L., Baldacci S., Angino A., Pistelli F., Di Pede F., Viegi G. (2004). Indoor exposures and acute respiratory effects in two general population samples from a rural and an urban area in Italy. J. Expo. Anal. Environ. Epidemiol..

[124] Batterman S., Jia C., Hatzivasilis G. (2007). Migration of volatile organic compounds from attached garages to residences: A major exposure source. Environ. Res..

[125] Rojas-Bracho L., Suh H.H., Koutrakis P. (2000). Relationships among personal, indoor, and outdoor fine and coarse particle concentrations for individuals with COPD. J. Expo. Anal. Environ. Epidemiol..

[126] Abt E., Suh H.H., Allen G., Koutrakis P. (2000). Characterization of indoor particle sources: A study conducted in the metropolitan Boston area. Environ. Health Perspect..

[127] Klepeis N.E., Bellettiere J., Hughes S.C., Nguyen B., Berardi V., Liles S., Obayashi S., Hofstetter C.R., Blumberg E., Hovell M.F. (2017). Fine particles in homes of predominantly low-income families with children and smokers: Key physical and behavioral determinants to inform indoor-air-quality interventions. PLoS ONE.

[128] Gillespie-Bennett J., Pierse N., Wickens K., Crane J., Nicholls S., Shields D., Boulic M., Viggers H., Baker M., Woodward A. (2008). Sources of nitrogen dioxide (NO2) in New Zealand homes: Findings from a community randomized controlled trial of heater substitutions. Indoor Air.

[129] Paulin L.M., Diette G.B., Scott M., McCormack M.C., Matsui E.C., Curtin-Brosnan J., Williams D.L., Kidd-Taylor A., Shea M., Breysse P.N. (2014). Home interventions are effective at decreasing indoor nitrogen dioxide concentrations. Indoor Air.

[130] Colton M.D., MacNaughton P., Vallarino J., Kane J., Bennett-Fripp M., Spengler J.D., Adamkiewicz G. (2014). Indoor air quality in green vs. conventional multifamily low-income housing. Environ. Sci. Technol..

[131] Vardoulakis S., Dimitroulopoulou C., Thornes J., Lai K.M., Taylor J., Myers I., Heaviside C., Mavrogianni A., Shrubsole C., Chalabi Z. (2015). Impact of climate change on the domestic indoor environment and associated health risks in the UK. Environ. Int..

[132] Coombs K.C., Chew G.L., Schaffer C., Ryan P.H., Brokamp C., Grinshpun S.A., Adamkiewicz G., Chillrud S., Hedman C., Colton M. (2016). Indoor air quality in green-renovated vs. non-green low-income homes of children living in a temperate region of US (Ohio). Sci. Total Environ..

[133] Lung S.-C.C., Mao I.F., Liu L.-J.S. (2007). Residents’ particle exposures in six different communities in Taiwan. Sci. Total Environ..

[134] McCormack M.C., Breysse P.N., Hansel N.N., Matsui E.C., Tonorezos E.S., Curtin-Brosnan J., Williams D.L., Buckley T.J., Eggleston P.A., Diette G.B. (2008). Common household activities are associated with elevated particulate matter concentrations in bedrooms of inner-city Baltimore pre-school children. Environ. Res..

[135] Cortez-Lugo M., Moreno-Macias H., Holguin-Molina F., Chow J.C., Watson J.G., Gutiérrez-Avedoy V., Mandujano F., Hernández-Ávila M., Romieu I. (2008). Relationship between indoor, outdoor, and personal fine particle concentrations for individuals with COPD and predictors of indoor-outdoor ratio in Mexico city. J. Expo. Sci. Environ. Epidemiol..

[136] Romagnoli P., Balducci C., Perilli M., Vichi F., Imperiali A., Cecinato A. (2016). Indoor air quality at life and work environments in Rome, Italy. Environ. Sci. Pollut. Res. Int..

[137] Byun H., Bae H., Kim D., Shin H., Yoon C. (2010). Effects of socioeconomic factors and human activities on children’s PM_10_ exposure in inner-city households in Korea. Int. Arch. Occup. Environ. Health.

[138] Kovesi T., Creery D., Gilbert N.L., Dales R., Fugler D., Thompson B., Randhawa N., Miller J.D. (2006). Indoor air quality risk factors for severe lower respiratory tract infections in Inuit infants in Baffin Region, Nunavut: A pilot study. Indoor Air.

[139] Raaschou-Nielsen O., Sørensen M., Hertel O., Chawes B.L., Vissing N., Bønnelykke K., Bisgaard H. (2011). Predictors of indoor fine particulate matter in infants’ bedrooms in Denmark. Environ. Res..

[140] Pavilonis B.T., Anthony T.R., O’Shaughnessy P.T., Humann M.J., Merchant J.A., Moore G., Thorne P.S., Weisel C.P., Sanderson W.T. (2013). Indoor and outdoor particulate matter and endotoxin concentrations in an intensely agricultural county. J. Expo. Sci. Environ. Epidemiol..

[141] García Algar O., Pichini S., Basagaña X., Puig C., Vall O., Torrent M., Harris J., Sunyer J., Cullinan P. (2004). Concentrations and determinants of NO2 in homes of Ashford, UK and Barcelona and Menorca, Spain. Indoor Air.

[142] Massey D., Kulshrestha A., Masih J., Taneja A. (2012). Seasonal trends of PM_10_, PM_5.0_, PM_2.5_ & PM_1.0_ in indoor and outdoor environments of residential homes located in North-Central India. Build. Environ..

[143] Vanker A., Barnett W., Nduru P.M., Gie R.P., Sly P.D., Zar H.J. (2015). Home environment and indoor air pollution exposure in an African birth cohort study. Sci. Total Environ..

[144] Lawrence A.J., Masih A., Taneja A. (2005). Indoor/outdoor relationships of carbon monoxide and oxides of nitrogen in domestic homes with roadside, urban and rural locations in a central Indian region. Indoor Air.

[145] Topp R., Cyrys J., Gebefügi I., Schnelle-Kreis J., Richter K., Wichmann H.E., Heinrich J. (2004). Indoor and outdoor air concentrations of BTEX and NO_2_: Correlation of repeated measurements. J. Environ. Monit..

[146] Khoder M., Shakour A., Farag S.A., Awad A. (2000). Indoor and outdoor formaldehyde concentrations in homes in residential areas in Greater Cairo. J. Environ. Monit..

[147] Su F.C., Mukherjee B., Batterman S. (2013). Determinants of personal, indoor and outdoor VOC concentrations: An analysis of the RIOPA data. Environ. Res.

[148] Brown K.W., Sarnat J.A., Suh H.H., Coull B.A., Spengler J.D., Koutrakis P. (2008). Ambient site, home outdoor and home indoor particulate concentrations as proxies of personal exposures. J. Environ. Monit..

[149] Nazariah S., Jalaludin J., Md Akim A. (2013). Interleukin-6 via Sputum Induction as Biomarker of Inflammation for Indoor Particulate Matter among Primary School Children in Klang Valley, Malaysia. Glob. J. Health Sci..

[150] Brauer M., Hrubá F., Mihalíková E., Fabiánová E., Miskovic P., Plziková A., Lendacká M., Vandenberg J., Cullen A. (2000). Personal exposure to particles in Banská Bystrica, Slovakia. J. Expo. Anal. Environ. Epidemiol..

[151] Simons E., Curtin-Brosnan J., Buckley T., Breysse P., Eggleston P.A. (2007). Indoor environmental differences between inner city and suburban homes of children with asthma. J. Urban Health.

[152] Hulin M., Caillaud D., Annesi-Maesano I. (2010). Indoor air pollution and childhood asthma: Variations between urban and rural areas. Indoor Air.

[153] Chatzis C., Alexopoulos E.C., Linos A. (2005). Indoor and outdoor personal exposure to benzene in Athens, Greece. Sci. Total Environ..

[154] Jones J., Stick S., Dingle P., Franklin P. (2007). Spatial variability of particulates in homes: Implications for infant exposure. Sci. Total Environ..

[155] Nasir Z., Colbeck I. (2013). Particulate pollution in different housing types in a UK suburban location. Sci. Total Environ..

[156] Russo E.T., Hulse T.E., Adamkiewicz G., Levy D.E., Bethune L., Kane J., Reid M., Shah S.N. (2015). Comparison of indoor air quality in smoke-permitted and smoke-free multiunit housing: Findings from the Boston Housing Authority. Nicotine Tob. Res..

[157] King B.A., Travers M.J., Cummings K.M., Mahoney M.C., Hyland A.J. (2010). Secondhand smoke transfer in multiunit housing. Nicotine Tob. Res..

[158] World Health Organization (2000). Air Quality Guidelines for Europe.

[159] World Health Organization (2006). Air Quality Guidelines. Global Update 2005.

[160] Vardoulakis S., Kettle R., Cosford P., Lincoln P., Holgate S., Grigg J., Kelly F., Pencheon D. (2018). Local action on outdoor air pollution to improve public health. Int. J. Public Health.

[161] Baxter L.K., Clougherty J.E., Laden F., Levy J.I. (2007). Predictors of concentrations of nitrogen dioxide, fine particulate matter, and particle constituents inside of lower socioeconomic status urban homes. J. Expo. Sci. Environ. Epidemiol..

[162] Rotko T., Koistinen K., HÄNninen O., Jantunen M. (2000). Sociodemographic descriptors of personal exposure to fine particles (PM_2.5_) in EXPOLIS Helsinki. J. Expo. Sci. Environ. Epidemiol..

[163] Shrubsole C., Macmillan A., Davies M., May N. (2014). 100 Unintended consequences of policies to improve the energy efficiency of the UK housing stock. Indoor Built Environ..

[164] World Health Organization (2011). Health in the Green Economy: Health Co-Benefits of Climate Change Mitigation-Housing Sector.

[165] Asikainen A., Carrer P., Kephalopoulos S., de Fernandes E.O., Wargocki P., Hänninen O. (2016). Reducing burden of disease from residential indoor air exposures in Europe (HEALTHVENT project). Environ. Health.

[166] Kelly F.J., Fussell J.C. (2019). Improving indoor air quality, health and performance within environments where people live, travel, learn and work. Atmos. Environ..

[167] US EPA (2020). Improving Indoor Air Quality. Indoor Air Quality (IAQ). US Environmental Protection Agency. https://www.epa.gov/indoor-air-quality-iaq/improving-indoor-air-quality.

[168] RCPCH & RCP (2020). The inside story: Health effects of indoor air quality on children and young people. Royal College of Paediatrics and Child Health & Royal College of Physicians. https://www.rcpch.ac.uk/resources/inside-story-health-effects-indoor-air-quality-children-young-people.

[169] Vardoulakis S., Crawford J., Davis A., Steinle S., Sleeuwenhoek A., Galea K., Dixon K. (2019). Indoor Exposure to Selected Air Pollutants and Associated Health Effects–a Global Review.

