# Supplementary material for: Indoor Exposure to Selected Air Pollutants in the Home Environment: A Systematic Review
Source: Int J Environ Res Public Health. 2020 Dec 2;17(23):8972. doi: 10.3390/ijerph17238972 (PMC7729884; doi:10.3390/ijerph17238972)
Supplement: Supplementary file 1 [file ijerph-17-08972-s001.pdf]

International Journal of
Environmental Research and Public Health

MDPI

Review

Supplementary Materials: Indoor Exposure to Chemical Air Pollutants in the Home Environment: A Systematic Review

Sotiris Vardoulakis [1,2], Evanthia Giagloglou [1], Susanne Steinle [1], Alice Davis [1], Anne Sleeuwenhoek [1], Karen S. Galea [1], Ken Dixon [1] and Joanne O. Crawford [1,3]

1 Institute of Occupational Medicine (IOM), EH14 4AP Edinburgh, UK; eva.giagloglou@iom-world.org (E.G.); susanne.steinle@iom-world.org (S.S.); alice.davis@iom-world.org (A.D.); anne.sleeuwenhoek@iom-world.org (A.S.); karen.galea@iom-world.org (K.S.G.); ken.dixon@iom-world.org (K.D.); joanne.crawford@vuw.ac.nz (J.O.C.)

2 National Centre for Epidemiology and Population Health, Research School of Population Health, Australian National University, ACT 2601 Canberra, Australia

3 Faculty of Health, Victoria University of Wellington, 6410 Wellington, New Zealand

* Correspondence: sotiris.vardoulakis@anu.edu.au

Search strategy

We extracted the following information from all eligible papers: type of study (e.g., monitoring or modelling); research question; dwelling type (detached, semi-detached, terraced, flat/floor); dwelling distance from outdoor sources (main road, industrial source, etc.); city, region, and country; occupant characteristics (age group, socioeconomic status, health status); study design (cross sectional, case control, before-after intervention, etc.); indoor sources (cooking, heating, tobacco smoking, consumer products, furnishings, etc.); pollutants monitored (e.g., $PM_{2.5}$, NO_2); sampling location (bedroom, kitchen, etc.); time, day and/or season of measurements; health outcome examined (e.g., asthma) and assessment method (self-reported, spirometry, etc.); reported effects of indoor air pollution on health; reported indoor exposure levels; reported determinants of indoor exposure (e.g., ventilation, building characteristics, indoor sources, occupancy patterns); reported evidence gaps; measurement methods (active sampling, passive sampling, equipment used, etc.); time duration of measurements; number of measurements taken.

On completion of data extraction, we rejected the poorer-quality papers (i.e., those based on one measurement or a single house), as well as those which did not comply with the following quality assessment criteria:

(a) Measurement method—is it reported and is it a recognised method or equipment?

(b) Measurement duration—are measurements carried out for at least 6 hours?

(c) Number of measurements—is this listed within the paper?

(d) Location where the sampler was placed—is this noted within the paper?

From the data extracted, evidence tables were prepared to aid the assessment process and to provide accessible documentation of the evidence on which the review is based.

Search terms

((ab(house* OR bungalow* OR flat* OR chalet* OR tenement* OR domicile* OR home* OR “terraced house*” OR (domestic AND propert*) OR (domestic AND dwelling*) OR (domestic AND building*)) AND "indoor air" AND (“air pollution” OR “air quality” OR “particulate matter” OR “Nitrogen Dioxide” OR NO2 OR Ozone OR O3 OR “Sulphur Dioxide” OR SO2 OR “ultrafine particles” OR VOCs OR "volatile organic compounds" OR Formaldehyde OR Benzene OR Naphthalene OR PAH OR Trichloroethylene OR ("Carbon Monoxide" NOT poison*)) AND (review OR "observational stud*") AND (exposure OR monitor* OR sampl* OR measure*) NOT ("biological

pollutant*" OR "CO acute poisoning" OR ("biomass burning" AND rural) OR asbestos OR radon OR methane))) and (pd(20000101-20171128)) and (peer(yes)) and (stype.exact("Scholarly Journals" OR "Reports") AND la.exact("English"))

Table S1. Summary of eligible study findings. Information on measurement methods is available in Vardoulakis et al. (2019) [169]. (TVOC: total volatile organic compounds; TPAH: total polycyclic aromatic hydrocarbons; PM: particulate matter; UFP: ultrafine particles; TSP: total suspended particles; BC: black carbon; EC: elemental carbon; SBS: sick building syndrome; GM: geometric mean.)

Reference	Population and dwelling type	City/region; country	Indoor pollution sources	Pollutants	Sampling location	Month/ season and year	Indoor exposure levels	Determinants of indoor exposure
**Abt et al. 2000 [126]**	Not recorded	Boston area; USA	Cooking, heating, carpet, cleaning	PM2.5–10, PM0.02–0.1, PM0.1–0.5, PM0.7–2.5	Adjacent to area where the majority of activities occurred (i.e., kitchen and living room)	March 1996–June 1996	Median concentrations (μg/m^3) measured using Harvard impactors:<br>PM2.5: 11.6<br>PM10: 17.8<br>PM2.5–10: 4.3<br>Median concentrations (μg/m^3) measured using SMPS/APS:<br>PM0.02–0.1: 0.33<br>PM0.1–0.5: 5.5<br>PM0.7–2.5: 2.2<br>PM2.5–10: 2.7	Seasons, oven cooking, frying, toasting, cleaning, number of occupants, air exchange rates
**Adgate et al. 2004 [80]**	153 households (single family detached, single family attached, apartment, other)	Minneapolis USA	Smoking, room deodorisers	15 VOCs	Room where child spent most of time while awake	Winter, January 2000–February 2000; spring April 2000–May 2000	Median concentrations in winter and spring (μg/m^3):<br>benzene: 2.2, 2.1<br>ethylbenzene: 1.0, 1.0<br>d-limonene: 28.6, 21.2<br>α-pinene: 2.4, 2.4<br>β-pinene: 2.5, 1.5<br>toluene: 8.2, 8.9<br>m,p-xylene: 3.7, 3.3<br>o-xylene: 1.2, 1.1	Smoking, cleaning supplies
**Adgate et al. 2004 [72]**	248 households (single-family homes, apartments, mobile home)	Minnesota; USA	Smoking, central air conditioning	VOCs	Room where the family spent most time	May 1997–September 1987	Median concentrations (μg/m^3):<br>benzene: 4.6<br>m,p-xylene: 7.9<br>o-xylene: 2.9<br>toluene: 23.4	Not reported
**Alexopoulos et al. 2006 [77]**	50 households	Athens; Greece	Smoking	Toluene, xylene	Not reported	September 1997–September 1998	Mean concentrations (μg/m^3):<br>toluene: 49.2<br>xylene: 43.7	Location (centre or suburb), proximity to busy road and proximity to gasoline station, heating mode, recent painting, type/floor of

								house, use of oil and natural gas ovens, wind speed, ventilation
**Batterman et al. 2007 [124]**	15 households (single-family houses)	Ann Arbor, Ypsilanti; USA	Garage, heating/cooling system, other activities	VOCs	Varies from house to house	April–July	Mean concentrations (μg/m³): VOCs: 12 benzene: 2.0 toluene: 26.5 ethylbenzene: 2.3 m,p-xylene: 8.3 o-xylene: 2.9 naphthalene: 8.3 CO2: 615.58 ppm	Configuration of house, attached garage, ventilation, air exchange rates, heating/cooling system
**Batterman et al. 2012 [116]**	288 households	Ann Arbor (AA), Ypsilanty (YP), Dearborn (DB), Detroit (DT); USA	Repellent, deodorizer, exhaust and evaporative emissions in garages, tobacco smoke, wood combustion, incense burning	Naphthalene, environmental tobacco smoke (ETS), 2,5-dimethyl furan, 3-ethynyl pyridine (3-EP)	Living room, child's bedroom	2004–2005 except DT (March 2009–October 2010)	Naphthalene mean concentration (μg/m³): Overall: 5.4 AA: 3.5 YP: 5.6 DB: 2.3 DT: 7.8	Not reported
**Baxter et al. 2007 [159]**	43 households (single-family, multifamily, apartment)	Boston; USA	Cleaning, gas stove, smoking, humidifier, candle, cooking	NO2, PM2.5, EC	Main living area	Multiple seasons from 2003–2005	Mean concentrations: NO2: 19.6 ppb PM2.5: 20.3 μg/m³ EC: 0.57 x 10^{-5} m^{-1}	Cooking duration, gas stove usage, occupant density, humidifier use, candle use, cleaning, natural ventilation
**Belanger et al. 2006 [63]**	242 households	Connecticut, Massachusetts; USA	Gas stoves, gas dryers, smoking, mould	NO2	Not recorded	November–March; April–October	NO2 mean concentration (ppb): Homes with electric ranges: 8.6 Homes with gas ranges: 25.9	Use of gas stoves, season, single or multifamily housing, smoking

**Belanger et al. 2013 [57]**	1342 households	Connecticut, Massachusetts; USA	Gas cooking, smoking	NO2	Dayroom, child's bedroom	2006–2009 All seasons	NO2 overall mean: 10.6 ppb	Living in multifamily housing, ethnicity, level of education, stove type
**BéruBé et al. 2004 [42]**	6 households (urban, suburban, rural)	Wales, Cornwell; UK	Smoking, pets cleaning restrictions, heating	PM10	Kitchen, living room, bedroom	All four seasons (1998–1999)	PM10 overall annual mean concentration: 25.52 μg/m^3	Smoking, cleaning, temperature, relative humidity
**Bhangar et al. 2011 [46]**	7 (single-family, detached houses)	Alameda County; USA	Heating, natural gas furnace, electric appliances, cleaners, household activities, cooking, candles	UFP, O3, NO, CO2, CO	Central location, e.g., living or dining room; second monitor in different location	All four seasons, November 2007–February 2009	PNC GM: 14500 #/cm3 PNC AM: 17000 #/cm3	Use of gas appliances, electric stove or oven use, toaster use, ironing clothes, microwave use, using candles, terpene-based cleaning product use, vacuum cleaning, sweeping, clothes washing, other electric appliances, non-terpene cleaning product use
**Blanc et al. 2005 [7]**	226 households	California; USA	Pets, heating type, carpets	CO, CO2, NO2, formaldehyde, acrolein, VOCs, allergens, endotoxins	Kitchen, living room, bedroom	1992, 1996, 1999	Mean concentrations: CO2: 760 ppm CO: 0.3 ppm NO2: 13 ppb formaldehyde: 15 ppb	House dust, presence of pets, air filtration, wall dampness, room humidity, gas stoves, wood heating
**Brauer et al. 2000 [150]**	49 households (apartment buildings, semi-detached houses, detached houses)	Banska Bystrica; Slovakia	Not reported	PM10, PM2.5, SO4	Room people reported spending most time in when not sleeping	Summer, June 1997–September 1997; winter, November	Mean concentrations in summer, winter (μg/m^3): PM10: 79, 66 PM2.5: 55, 53 SO4: not reported, 4.6	Season, location

						1997–March 1998		
**Brown et al. 2008 [148]**	25 households (apartments)	Boston; USA	Cooking, gas stove, heating system, candles	SO4/-2, PM2.5, EC	Living room	Winter, November 1999–January 2000; summer, June 2000–July 2000	GM concentrations (μg/m^3): SO4/-2: winter: 1.5 summer: 3.1 PM2.5: winter: 10.1 summer: 12.0 EC: winter: 1.9 summer: 1.5	Infiltration rate, season, ventilation, cleaning, cooking, candle burning, housing characteristics, ambient concentrations, heating system
**Brown et al. 2014 [24]**	10 households (4 with OWF vs. 6 controls)	Connecticut ; USA	Outdoor wood furnaces (OWF)	PNC (PM2.5) PNC (PM0.5)	Room exposed to wood smoke and frequented by family (i.e., bedroom, living room or study)	Winter (January–March) 2010	PM2.5 mean (#/m3) OWF exposed: 0.302 x10^6 OWF control: 0.0718 x10^6	Vacuuming, cooking and smoking
**Byun et al. 2010 [137]**	50 households (mostly apartment style)	Ansan-city (AS), Siheung-city (SH), Seongnam-city (SN); Korea	Cooking, cleaning, smoking	PM10	Living room and children's bedroom	July 2008–September 2008	PM10 mean concentrations (μg/m^3): Living rooms: 45.3 Children's bedrooms: 45.9	Region, parental education, floor of residence, building age, average monthly household expenses, number of children, cooking, cleaning, smoking
**Cao et al. 2005 [35]**	6 households (urban, rural, roadside)	Tsim Sha Tsui, Tseung Kwan O Kamtin, Tai Pol; Hong Kong, China	Smoking, cooking, cleaning	PM2.5, organic carbon, elemental carbon	Living room or bedroom	March 2004–April 2004	PM2.5 mean concentration: 56.7 μg/m^3	Housing near main road or construction site

**Chatzis et al. 2005 [153]**	50 households (detached house, flat/apartment)	Athens; Greece	Building materials, heating mode, solvents, adhesives, smoking	Benzene	Attached to volunteer's lapel and during night set in bedside table	September 1997–September 1998	Benzene concentration in six periods (μg/m^3): September: 13.4 December: 11.2 February: 10.2 April: 9.0 June: 5.4 September: 7.8	Season, wind speed, building materials, ventilation, proximity to busy road, smoking, heating mode, proximity to petrol station, house characteristics
**Chen et al. 2017 [43]**	1 students' dormitory (10th floor), 1 apartment (9th floor)	Beijing; China	Camphor pollution, cooking sources, outdoor pollution	PM2.5, PAHs	Not reported	Four seasons, March 2015–January 2016	Mean concentrations: PM2.5: 43.8 μg/m^3 PAHs: 36.9 ng/m^3	Seasons, outdoor air, use of naphthalene
**Cheng et al. 2016 [71]**	40 households (freestanding, single-story suburban residences)	Melbourne; Australia	Building materials, subfloor material, floor covering, domestic chemicals, cooking	VOCs, CO, CO2, PM2.5, PM10, NO2, O3	Living room	Winter/spring/summer 2008 (August–December), summer/autumn 2009 (January–May)	Mean concentrations (μg/m^3): benzene: 1.3 toluene: 10.7 ethylbenzene: 1.2 p-xylene: 2.9 m-xylene: 1.2 o-xylene: 2.2 formaldehyde: 16.4 acetaldehyde: 7.6 naphthalene: 1.5	Air conditioning, ventilation, presence of domestic chemicals, type of floor covering, dwelling age, proximity to major roads, outdoor air quality
**Chi et al. 2016 [53]**	19 households	Taiwan	Road traffic, cooking, smoking	PM2.5, PM10	Living room, bedroom, kitchen	March 2014–May 2016	Mean concentrations (μg/m^3): PM2.5: bedroom 104.7 kitchen 119.0 living room 104.7 PM10: bedroom 104.6 kitchen 123.4 living room 114.6	Not recorded
**Chin et al. 2014 [73]**	126 Homes	Detroit; USA	Smoking, air fresheners, moth crystals, building materials, solvents, inks, coatings, paint, perfume, oils,	56 VOCs in total. Priority VOCs: benzene, toluene,	Child's bedroom main living area	March 2009–September 2010 All seasons	Mean concentrations (μg/m^3): benzene: 2.27 toluene: 11.62 ethylbenzene: 1.72 m/p-xylene: 6.34	Air exchange rate, season, in-house airflow and mixing, attached garage, age and

			adhesives, varnish, glues, pesticides	ethylbenzene, m/p-xylene, o-xylene, naphthalene			o-xylene: 2.02<br>naphthalene: 7.88	condition of the house, outdoor sources, e.g., vehicle emissions
**Cibella et al. 2015 [61]**	323 households (detached house /condominium)	Palermo; Italy	Gas appliances, smoking, road traffic	NO2	Main living area	Spring (April–May) and winter (January–February)	NO2 mean concentrations (μg/m^3):<br>spring: 31.9<br>winter: 32.2	Presence of gas appliances, second hand smoke, window opening, number of rooms, type of dwelling, floor of residence, number of years in property, crowding
**Cirillo et al. 2006 [114]**	30 households	Campania; Italy	heating, smoking	PAHs	Not reported	December 2004	PHA mean concentrations (ng/m^3):<br>urban homes: 2.5<br>rural homes: 4.1	Contamination of PAHs in food, smoking, type of heating,
**Clarisse et al. 2003 [100]**	61 households (flats with at least 3 separate rooms)	Paris and surrounds; France	Smoking	6 Aldehydes	Kitchen, living room, bedroom	March 2001–June 2001; September 2001–October 2001	GM concentrations (μg/m^3) in kitchens, living rooms, and bedrooms:<br>formaldehyde: 21.7, 24.3, 24.5<br>acetaldehyde: 10.1, 10.0, 10.2<br>pentanal: 5.7, 6.0, 6.4<br>hexanal: 20.5, 23.8, 25.5	Type of floor or wall coverings, refurbishment, building age, smoking, room type, ambient parameters
**Clougherty et al. 2011 [17]**	43 households (mainly multifamily units, three to four story residential buildings)	Boston and surrounds; USA	Cooking, cleaning, smoking, candle burning	NO2, PM2.5, BC	Main living area	Summer and winter, 2003–2005	Mean concentrations:<br>PM2.5: 20.1 μg/m^3<br>NO2: 19.1 ppb<br>BC: 0.58 x 10^{-5} m^{-1}	Indoor combustion, cleaning, resuspension, natural ventilation, exhaust fan use
**Colton et al. 2014 [130]**	61 households (43 conventional vs. 18 green, low-income public housing)	Boston; USA	Smoking, cooking, candles, air freshener	PM2.5, NO2, formaldehyde	Main living space	2012–2013 (season not recorded)	Geometric mean (μg/m^3):<br>PM2.5 (total): 12.9<br>PM2.5 (conv.): 15.1<br>PM2.5 (green): 8.9<br>NO2 (total): 46.0	Cooker type, smoking policy, air exchange rate

							NO2 (conv.): 63.2<br>NO2 (green): 21.4<br>formaldehyde (total): 10.2<br>formaldehyde (conv.): 9.4<br>formaldehyde (green): 12.1	
**Coombs et al. 2016 [132]**	42 households (green units, non-green units)	Cincinnati, Ohio; USA	Building materials, renovation products, toilet deodorant, air freshener, nail polish, mothballs	PM2.5, BC, UFP, TVOC, formaldehyde	Child's or parents' bedroom	Heating season, October–March; non-heating season, April–September	Median concentrations:<br>PM2.5: 41 μg/m³<br>BC: 0.98 μg/m³<br>UFP: 19000 #/cm3<br>TVOC: 1.28 μg/m³<br>formaldehyde: 24.6 μg/m³	Frequency of opening windows, occupants activities
**Cortez-Lugo et al. 2008 [135]**	38 households	Mexico City; Mexico	Carpeting, aerosol spray, tobacco smoke	PM2.5, PM10	Area of the house where participants spend most time (excluding kitchen)	February 2000–Nov 2000	Mean concentrations (μg/m³):<br>PM2.5: 32<br>PM10: 50	Carpeting, using aerosol spray, boiler location, smoking and natural ventilation
**Custódio et al. 2014 [41]**	4 homes (1 terraced house, 3 apartments)	Aveiro, São João da Madeira; Portugal	Cooking, smoking, pets, fireplace	PM10	Kitchen	January–March 2011, 2012	PM10 mean concentration:<br>71.9 μg/m³	Natural ventilation, smoking
**Cyrys et al. 2000 [59]**	405 households	Hamburg, Erfurt; Germany	Heating, cooking, smoking	NO2	Living room, bedroom	June 1995–November 1996	NO2 median concentrations (μg/m³):<br>Erfurt: 15<br>Hamburg: 17	Season, building materials, building age, type of heating, type of fuel used, gas cooking, ventilation, smoking
**Delfino et al. 2004 [52]**	Not recorded	Alpine, California; USA	Not recorded	PM2.5, PM10	Main living area	September 1999–October 1999; April 2000–June 2000	Mean concentrations (μg/m³):<br>PM2.5: 12.1<br>PM10: 30.3	Not recorded
**Delgado-Saborit et al. 2011 [68]**	100 households	London, West Midlands, rural South Wales; UK	Tobacco smoke, candle/ incense, fireplace, home activities	15 VOCs	Generally in the living room	May 2005–May 2007	Mean concentrations (μg/m³):<br>benzene: 1.97<br>toluene: 17.53<br>ethylbenzene: 1.74<br>naphthalene: 0.79<br>p-xylene: 1.7	Smoking, ventilation, use of cleaning products, building materials,

							m-xylene: 4.14<br>o-xylene: 2.02	garages connected to the main house, season
**Du et al. 2015 [82]**	61 households	Detroit, Michigan; USA	Solvents, household cleaners, air fresheners, smoking, gas-powered tool, adhesives, paints, lubricants, petrol, wood products, nail polish, perfume, moth balls, pesticides	Benzene, toluene naphthalene, limonene	Basement, living area	All seasons from August 2011–Dec 2011, and seasonal assessment spaced 2–3 months apart	Mean concentrations (μg/m^3)<br>benzene:<br>house 2.21<br>basement 2.98<br>toluene:<br>house 11.81<br>basement 21.75<br>naphthalene:<br>house 26.30<br>basement 17.19<br>limonene:<br>house 20.16<br>basement 16.60	Heating and cooling system, no. of windows, degree of wind sheltering, dimensions of rooms, basement, overall exterior dimensions, no. of floors, air flow rates, interzonal flows, smoking
**Ferrero et al. 2017 [79]**	352 households	Valencia; Spain	Heating, smoking, solvent-based paintings, redecoration activities	Benzene	Living room	2006–2007	Benzene mean concentration: 1.46 μg/m^3	Mothers age, type of heating, maternal country of birth, parental tobacco consumption, ventilation, seasons
**García Algar et al. 2004 [141]**	1421 households	Ashford; UK. Menorca, Barcelona; Spain	Cooking, gas combustion, smoking	NO2	Living room	November 1993–July 1995; 1996–1998	NO2 median concentrations (ppb):<br>Ashford: 5.79<br>Menorca: 6.06<br>Barcelona: 23.87	Gas cooking, smoking, heating mode, house location
**Gilbert et al. 2005 [102]**	59 households (trailer/mini home, single detached house, side-by-side duplex, other)	Prince Edward Island; Canada	Smoking, type of heating/heating fuel, wood stove use, varnishing, odours of fresh paint, new carpet, painting	Formaldehyde, acetaldehyde, acrolein, CO2	Not recorded	Winter 2002	GM concentrations:<br>formaldehyde: 33.2 μg/m^3<br>CO2: 850 ppm<br>acetaldehyde: 20.2 μg/m^3<br>acrolein: 1.0 μg/m^3	Smoking, air exchange rates, absolute humidity, building age
**Gilbert et al. 2006 [103]**	96 households (single-family dwellings, duplexes, triplexes)	Quebec City; Canada	Cooking appliances, wood burning, painting	NO2, formaldehyde	Living room	January 2005–April 2005	GM concentrations (μg/m^3):<br>formaldehyde: 29.5<br>NO2: 8.3	House type, air exchange rate, heating and cooking systems, garage, ventilation

								building age, heat distribution
**Gillespie-Bennett et al. 2008 [128]**	409 households	Bluff, Dunedin, Christchurch, Porirua, Hutt Valley; New Zealand	Heating type, smoking,	NO2	Living room and bedroom of child with asthma	Winter, June–September 2006	NO2 geometric mean concentrations: 11.4 μg/m^3	Heating type, smoking, ventilation, gas or electric stove use, open windows, gas or electric oven use
**Gillespie-Bennett et al. 2011 [66]**	349 households	Bluff, Dunedin, Christchurch, Porirua, Hutt Valley; New Zealand	Unflued gas heating	NO2	Living rooms	Winter, June 2006–September 2006	NO2 geometric mean concentration: 11.4 μg/m^3	heating systems, ambient air
**Gordian et al. 2010 [91]**	509 households (single-family homes with attached garage)	Anchorage; USA	gasoline-fuelled equipment, solvents and other items containing gasoline stored in the garage	VOCs	Primary living space	November 2008–April 2009	Median concentrations (ppb): benzene: 2.88 toluene: 7.34 ethylbenzene: 0.83 xylenes: 3.01	Small engines and gasoline stored in attached garages
**Guo et al. 2009 [89]**	100 households (houses, apartments and others)	Hong Kong; China	Building materials, furnishings	Formaldehyde, VOCs	Living room, if no living room, bedroom	Winter 2002	Mean concentrations (μg/m^3): formaldehyde: 112.3 VOCs: 46.1	Building age, pressed wood products, shoes inside the house, building type, number of people
**Gurley et al. 2013 [36]**	258 households	Dhaka; Bangladesh	Cooking fuel (biomass burning), smoking	PM2.5	Child's bedroom	May 2009–April 2010	PM2.5 mean concentration: 190 μg/m^3	Ventilation, smoking, solid fuel (biomass burning), season
**Hansel et al. 2008 [64]**	150 households (mostly row homes)	Baltimore; USA	Cooking, heating, cleaning, smoking, air purifier, candle, incense	NO2, PM2.5	Child's bedroom	All seasons	Mean concentrations: NO2: 30.0 ppb PM2.5: 40.3 μg/m^3	Gas stove/heater use, sweeping, use of a space heater or oven, season, incense/ candle burning, natural ventilation,

								smoking, house location (near street or parking)
**Hansel et al. 2013 [56]**	84 households	Baltimore; USA	SHS, combustion sources	PM2.5, NO2	Bedroom, main living area	Baseline, 3 months, 6 months	PM2.5 mean (μg/m³):<br>bedroom: 11.4<br>living area: 12.2<br>NO2 mean (ppb):<br>bedroom: 10.8<br>living area: 12.2	Room type, ventilation
**Harrison et al. 2009 [84]**	100 households (houses, flats)	London, Birmingham, West midlands, South Wales; UK	Solvents, cleaning, heating, cooking, redecorating, air fresheners, glue	VOCs, PAHs	Living room	All seasons, May 2005–May 2007	GM concentrations (μg/m³):<br>benzene: 1.5<br>toluene: 10.88<br>ethylbenzene: 1.17<br>p-xylene: 1.03<br>m-xylene: 2.55<br>o-xylene: 1.29<br>naphthalene: 0.52	Outdoor air, heating, cooking, redecorating, using air fresheners, smoking, time spent in garage
**Héroux et al. 2010 [121]**	145 households	Regina; Canada	New furniture, heating, air conditioning, cooking/cooking appliances, smoking, candles, perfume, hairspray	PM2.5, PM10–2.5, EC/OC, NO2, O3, CO, VOCs, and 43 aldehydes	Family or living room	Winter, January 2007–March 2007; summer, July 2007–August 2007	GM concentrations (μg/m³) in summer, winter:<br>formaldehyde: 31.08, 23.39<br>benzene: 1.28, 1.44<br>toluene: 11.26, 8.40<br>O3: 0.12, n/a<br>NO2: 8.51, n/a<br>PM2.5: 6.43, 5.46<br>acetaldehyde: 10.10, 9.74	Season, ventilation, new furniture/rug, attached garage, building age, off-gassing, air exchange rate, cooking with oil, smoking, gas stoves, using candles or perfume or hair spray
**Hu et al. 2017 [32]**	60 households	Harbin, Dalian, Beijing, Shanghai, Wuhan and Changsha; China	Outdoor pollution, redecoration, dampness, pets, low cleaning frequency of quilt	Acetaldehyde, PM2.5, formaldehyde, SVOC, TVOC	Child's bedroom, living room	Winter 2013	PM2.5 mean concentrations (μg/m³):<br>Beijing: 71<br>Harbin: 60<br>Dalian: 36.5<br>Formaldehyde highest indoor concentrations (μg/m³):<br>Beijing: 42.6<br>Shanghai: 31.1<br>Wuhan: 45.2<br>Changsha: 11.4	Time spent in living room and child's bedroom, poor natural ventilation, outdoor pollution

**Hulin et al. 2010 [152]**	114 households	Clermont-Ferrand; France	Not applicable to this paper	NO2, PM2.5, VOCs	Living room	Summer and winter (urban), summer (rural)	Median concentrations (μg/m^3): NO2: 10.5 PM2.5: 10.6 formaldehyde: 19.2 acetaldehyde: 13.6 benzene: 1.8 toluene: 20.2 ethylbenzene: 2.9 xylenes: 10.3	Season, urban versus rural location, ventilation, way of living, outdoor pollution
**Jafta et al. 2017 [122]**	114 households (houses, apartments, and informal shacks)	Durban; South Africa	Cooking, smoking, incense	NO2, PM10, SO2	Living room or sleeping area	Warm season, September–May; cold season June–August	Mean concentrations (μg/m^3): NO2: 19 PM10: 64 SO2: 0.6	Type of housing, cooking fuel type, distance to roadway, ventilation, season
**Jones et al. 2007 [154]**	78 households	Not reported	Smoking, incense, pets, carpet, cooking	PM10, PM2.5, PM total	Living room and bedroom	Not reported	Mean concentrations in the living room at 0.2m and 1.4m from ground, and in bedroom (μg/m^3): PM: 20.6, 21.7, 20.5 PM10: 13.2, 14.3, 13.2 PM2.5: 7.8, 8.0, 7.5	Floor level, smoking, incense burning, presence of pets, carpet, distance between houses and major boulevards, separated kitchen, cooking time
**Jones et al. 2000 [29]**	9 households (roadside homes, urban flats, rural country houses)	Birmingham, Oxfordshire, Worcestershire; UK	Smoking	PM10, PM2.5, PM1	Ground floor	1997	I/O ratio greater than 1 for all sites	Cooking (electric appliance), cleaning, air exchange rates, ventilation
**Jung et al. 2012 [51]**	408 households	New York City; USA	Space heating	PM2.5, BC	In the room the child spent the majority of her/his time	October 2005–May 2011	PM2.5 median concentrations (μg/m^3): new wheeze group: 12.2 reference group: 13.8 BC median concentrations (μg/m^3): new wheeze group: 1.42 reference group: 1.50	Traffic emissions penetrated from outside, heating, season
**Jurvelin et al. 2003 [87]**	15 households (single-family house, apartments, attached houses)	Helsinki; Finland	Home appliances, cosmetics, smoking	Carbonyls	Carried out by participants	May 1997–September 1997	Mean concentrations (μg/m^3): formaldehyde: 33.3 acetaldehyde: 10.1	Building products emissions, cleaning

**Karottki et al. 2015 [45]**	27 apartments	Copenhagen; Denmark	Not reported	PM2.5, PNC (10–300 nm)	Living room, bedroom	November 2010–May 2011	PM2.5 median concentrations (µg/m^3): living room: 6.3 bedroom: 6.3 PNC median concentration: 7100/cm3	Not reported
**Kattan et al. 2007 [65]**	469 households	Baltimore, Bronx, Chicago, Cleveland, Detroit, New York, St Louis, Washington DC; USA	Gas stove, smoking	NO2	Child's bedroom	February–July	NO2 median concentration: 29.8 ppb	Gas/ electric stove, location (city), season (average monthly temp), month, smoking, presence of stove vents
**Kennedy et al. 2009 [112]**	1 household (9 km from the central business district)	Brisbane; Australia	Intermittent wood smoke	PAHs	Not reported	June–August 2007	Indoor concentrations (µg/m^3): fluorene: <0.03 phenanthrene: 1.2 fluoranthene: 0.45 pyrene: 0.25 benz(a)anthracene: <0.03 chrysene: 0.03	Intermittent wood smoke
**Khoder et al. 2000 [146]**	7 households (flats)	Greater Cairo; Egypt	Smoking, petroleum fuel	Formaldehyde	Kitchens, bedrooms, living rooms	Spring, March–May 1999; summer June–August 1999	Formaldehyde mean concentrations (µg/m^3): kitchens: 89 bedrooms: 100 living rooms: 100	Building age, building materials, seasons, air temperature, relative humidity, smoking
**King et al. 2010 [157]**	30 households (multiunit housing)	Buffalo; USA	Smoking, cooking, pyrolysis (candle burning or non-tobacco smoking event), electrical appliances	PM2.5	Living room, shared hallway between participating units	July 2008–August 2009	Median concentrations (µg/m^3): smoke-permitted units: 20.2 smoke-free units: 8.3 hallways: 16.6	Smoking, rest not discussed
**Klepeis et al. 2017 [127]**	290 households (mix of detached houses, apartment/ condo, townhouse, duplex, trailer/mobile home)	San Diego, California; USA	Smoking, cooking and cooling fuel, heating (wood or gas), cleaning, using aerosol spray products, candles or incense	PM0.5–2.5 as PNC	Room closest to usual cigarette smoking location (as reported by participants)	Not reported	PNC range of observed weekly mean: 556– 28400 counts per 0.01 cubic feet	Ventilation activities, housing characteristics, particle generating activities

**Kliucininkas et al. 2011 [75]**	Location 1: 3rd floor, 5 storey university dormitory Location 2: 1st floor of a university building	Kaunas; Lithuania	Not reported	PM2.5, PM10, PM4, PAHs, VOCs	Location 1: indoor site inside a room, 3rd floor Location 2: storage facility, 1st floor of a university building	Winter and spring, January–February 2009; March–April 2009	Mean concentrations (μg/m^3): PM2.5: 29.57 PAH: 53.9 naphthalene: 0.12 ethylbenzene: 0.22 benzene: 0.63 toluene: 3.0 m-p-o-xylenes: 1.13	Season, traffic intensity, heating system
**Kornartit et al. 2010 [58]**	60 households	Hertfordshire; UK	Cooking, smoking	NO2	Living room, bedroom, kitchen	Winter 2000, summer 2001	NO2 mean concentration (ppb): bedrooms: 9.1 living rooms: 10.5 kitchens: 13.1	Gas cooking, cooking with electric cookers, smoking
**Kovesi et al. 2006 [138]**	20 households (single story, raised above ground level)	Nunavut; Canada	Tobacco smoke, furnaces and fuel type	CO2, NO2, PM	Not reported	January 2003–March 2003 (extremely cold weather)	Mean concentrations: CO2: 1201 ppm NO2: 5.6 ppb PM: 167872 per cubic foot	Nicotine concentration, number of occupants in the home, ventilation rates
**Kulshreshtha et al. 2008 [39]**	5 households (flats, independent houses)	Delhi; India	Household fuel	PM10, PM2.5, PM1.0, CO2, CO, SO2, NOx	Kitchen	April 2004–September 2004; December 2004–February 2005	Mean concentrations: PM10: 386 μg/m^3 PM2.5: 233.33 μg/m^3 CO2: 847 ppb NOx: 144 μg/m^3 CO: 4 mg/m^3 SO2: 133 μg/m^3	Usage of gas and kerosene stoves, seasons
**Kwon et al. 2015 [94]**	257 households with infants	Seoul, Cheonan, Ulsan regions; Korea	Pet, air freshener	TVOC	Infant's bedroom	Not reported	TVOC mean concentration: 174.7 μg/m^3	Location (urban, rural, industrial), smoking
**Lai et al. 2010 [33]**	9 households	Guangzhou; China	Smoking, cooking fuel	PM 2.5	Middle of each residence	June 2003–July 2003	PM2.5 mean concentration: 47.4 μg/m^3	Age. Air condition, natural ventilation, remodelling, LPG cooking fuel, smoking
**Lai et al. 2004 [2]**	50 households (units)	Oxford; UK	Smoking, cooking, heating, glue, paint	PM2.5, VOC, NO2, CO	Unspecified	December 1998–February 2000	GM concentrations (μg/m^3): PM2.5: 11.4	Gas cooking, smoking, glue usage, painting

							NO_2: 22.3<br>TVOC: 194	cultural practices, wind speed, renovation, ventilation
**Lajoie et al. 2015 [27]**	83 households (bungalow, cottage, twin duplex, triplex, quadruplex)	Quebec City; Canada	Not reported	Formaldehyde, NO_2, CO_2, VOCs	Child's bedroom	October 2008–June 2011	Formaldehyde: 37.0 μg/m^3<br>NO_2: 3.4 μg/m^3<br>CO_2: 905.5 ppm<br>PM2.5: 1.68 μg/m^3<br>toluene: 20.0 μg/m^3	Ventilation
**Lawrence et al. 2005 [144]**	15 households (grass/bamboo homes to high population brick houses)	Agra; India	Not reported	CO, NO_2, NO, NOx	Living room	Winter, October 2002–February 2003	Mean rural and urban concentrations (ppb):<br>CO: 1150, 1220<br>NO: 227.5, 385<br>NO_2: 230.75, 255<br>NOx: 458.25, 640	Building age, distance from road, traffic, green area, ventilation, cleaning, fuel type, heating source, frequency of incense burning, cooking oil, season
**Lee et al. 2002 [120]**	119 households	Southern California; USA	Combustion sources, cooking, heating	HONO, NO_2<br>O_3	Living room	April 1996–May 1996	Mean concentrations (ppb):<br>HONO: 4.6<br>NO_2: 28.0<br>O_3: 14.9	Air conditioner use, humidifier use, presence of gas range, natural ventilation
**Lee et al. 2014 [78]**	150 households	Seoul; Korea	Floor covering, renovations, new furniture/electronics, vacuuming, heating fuel, artificial air freshener, insecticides, air-purifying indoor plants, smoking	PM10, formaldehyde, CO_2, CO, NO_2, TVOC, benzene, toluene, ethyl-benzene, xylene, styrene	Living room, also child's room for VOCs	March 2008–April 2010	Mean (μg/m^3)<br>PM10: 66.3<br>formaldehyde: 75.6<br>TVOC: 648.2<br>benzene: 2.8<br>toluene: 95.3<br>ethylbenzene: 5.7<br>xylenes: 10.6<br>styrene: 3.5	House construction year, building type, ventilation, presence of mould, use of artificial air freshener
**Lévesque et al. 2001 [37]**	89 households (single-family homes, duplexes,	Quebec City; Canada	Wood heating, pets, mould, chemicals, ventilation	NO_2, PM10, CO,	Room where combustion appliances are	December 1995–March 1996	Mean concentrations in the basement and ground floor:<br>NO_2: 6.4 and 7.0 ppb	Room type, heating mode, cooking

	mobile home, townhouse, modular home)			formaldehyde	present and living room		Formaldehyde: 7.5 and 8.5 μg/m³ Large number of PM10 and CO were < detection limit	
**Li et al. 2016 [34]**	Heating season: 53 households; non-heating season: 54 households	Lanzhou; China	Cooking fuels (coal, gas and electricity), ingression of outdoor pollution	PM2.5	bedroom, kitchen	Heating season, February 2013–March 2013; non-heating season, September 2013	PM2.5 mean concentrations (μg/m³): Heating season: kitchen: 125 bedroom: 119 Non-heating season: kitchen: 80 bedroom: 80	Type of cooking fuel, space heating, food preparation (boiled, frying, etc.), cooking frequency, smokers present, window opening, air exchange between kitchen and bedroom
**Liu et al. 2001 [110]**	8 households (multistorey apartment/flats)	Hangzhou; China	Smoking, heating mode, cooking appliances	12 PAHs	Bedroom, kitchen, living room	Summer, July 1999; Autumn November 1999	PAHs geometric mean concentrations (ng/m3): summer: 6485 autumn: 9568	Combustion fuel type, cooking process, ventilation, use of mothballs, smoking
**Lovreglio et al. 2009 [97]**	59 households	Bari; Italy	New or restored furniture, smoking	Formaldehyde, acetaldehyde	Kitchen	January–June 2008	Mean concentrations (μg/m³): formaldehyde: 16.0 acetaldehyde: 10.7	New or restored furniture, smoking, natural ventilation, season
**Lu et al. 2011 [113]**	71 households	Hangzhou; China Shizuoka; Japan	Smoking, cooking, heating, mothballs, insect repellent	8 PAHs	Main living area	Summer (August 2006) and winter (January 2007)	Total PHA mean concentrations in Hangzhou (ng/m3): summer: 47 winter: 40 Total PAH mean concentration in Shizuoka (ng/m3): summer: 5.2 winter: 6.1	Presence of smoking, kerosene heating, using mothballs and insect repellent, cooking practices, house age and outdoor environment
**Lung et al. 2007 [133]**	45 households	Taipei, Taichung, Kaohsiung; Taiwan	Cooking, incense burning, smoking, housing conditions	PM10	Living room	November 1998–February 1999	PM10 GM concentration: 73.4 μg/m³	Cooking type and time, separated kitchen, incense burning,

								smoking, floor level, carpet, presence of pets, distance between house and major boulevards
**MacNeill et al. 2014 [18]**	50 households (detached, row house, duplex/ triplex).	Halifax; Canada	Cooking, heating, cleaning	PM2.5	Living room	Winter, January 2009–April; summer, June 2009–September 2009	PM2.5 median concentrations (μg/m^3): winter: 6.78 summer: 10.10	Cooking, candle use Wood fireplace use no. of windows open, attached garage building age, stove type, carpet, air exchange rates
**Madureira et al. 2016. [93]**	68 households (mainly apartments): 38 homes of children with asthma (cases), 30 with no asthma (controls)	Porto; Portugal	Floor and wall coverings, cooking, smoking, cleaning	VOCs, PM2.5, PM10, CO2	Child's bedroom	Winter, October–April	Cases vs. controls median concentrations (μg/m^3): PM2.5: 54 vs. 67 PM10: 56 vs. 71 d-limonene: 10.6 vs. 15.6	Cleaning frequency, living conditions, proximity to heavy traffic roads, attached garages, outdoor air flow rates, proximity to gasoline stations
**Marchand et al. 2006 [96]**	22 households (houses and flats)	Strasbourg; France	Building materials, smoking, unvented fuel-burning appliances	Formaldehyde, acetaldehyde, propionaldehyde, benzaldehyde, hexanal	Bedroom, living/dining room	June 2004–September 2004 September 2004–January 2005	Mean concentrations (μg/m^3) in living rooms, bedrooms: formaldehyde: 35.7, 46.1 acetaldehyde: 18.1, 18.2	Ventilation, smoking building age, building materials used (eg plywood floor), kerosene space heaters
**Maruo et al. 2010 [98]**	34 households (houses, apartments)	Kanto area; Japan	Air condition, smoking	Formaldehyde	Occupants chose location where they wished to know concentration	September 2007–October 2007	5–6-year-old houses had the highest concentration of formaldehyde followed by 3–5-year-old houses, 0–2-year-old houses. 16–30-year-old houses had the lowest concentration of formaldehyde. Mean formaldehyde concentrations	Temperature, window opening and air cleaners, renovations, smoking

							measured in apartments decrease as the time after renovation increased.	
**Masih et al. 2010 [118]**	10 households	Agra; India	Gas appliances, cooking, smoking, incense	PAHs, VOCs	Living room, kitchen	Winter, November 2006–February 2007	TPAHs mean concentration (ng/m^3): urban site: 1946.84 roadside site: 2824.87	Using gas utilities, cooking process, smoking, incense burning, using oil/ghee
**Massey et al. 2012 [142]**	10 households (roadside and urban locations)	Agra; India	Cooking smoking	PM10, PM5.0, PM2.5, PM1.0	Living room	October 2007–March 2009	Mean concentrations (µg/m^3) at roadside houses, urban houses: PM10: 247, 181 PM5.0: 211, 145 PM2.5: 161, 109 PM1.0: 111, 99	Natural ventilation, ventilation, cooking on stove, smoking, wind speed, humidity, waste management
**McCormack et al. 2008 [134]**	300 households (mainly row homes)	Baltimore; USA	Smoking, stove, oven, burned food, sweeping, vacuuming, air conditioning, air purifier, candles, incense	PM2.5, PM10	Child's bedroom	All seasons, September 2001–December 2003	Mean concentrations (µg/m^3): PM2.5: 39.5 PM10: 56.2	Smoking, stove use, oven use, sweeping, vacuuming, air conditioning, air purifier use, candles/incense burning, natural ventilation, space heating
**McCormack et al. 2009 [49]**	150 households	Baltimore; USA	Smoking, stove, oven, burned food, sweeping, vacuuming, air conditioning, air purifier, candles/ incense	PM2.5–10, PM 2.5	Child's bedroom	All seasons, September 2001–December 2003	Mean concentrations (µg/m^3): PM2.5–10: 17.4 PM2.5: 40.3	Season, smoking, cooking, air purifier use, candle/incense use, cleaning
**McCormack et al. 2011 [50]**	133 households (majority were row houses in close proximity to the roadway)	East Baltimore; USA	Smoking, cooking and cleaning activities	PM10–2.5, PM2.5	Child's bedroom	baseline, 3 months and 6 months so different seasons covered and grouped together here.	PM2.5–10 median concentrations (µg/m^3): children with nonatopic asthma: 13.4 children with atopic asthma: 11.6 PM2.5 median concentrations (µg/m^3): children with non-atopic asthma: 35.7 children with atopic asthma: 27.6	Not applicable to this paper

**McNamara et al. 2013 [25]**	50 households	Missoula; USA	Wood stove, pets	PM2.5, PM10–2.5	Living area	2 winters, 7 homes: November 2010–March 2011 43 homes: November 2011–March 2012	Mean concentrations (μg/m³): PM10–2.5: 12.9 PM2.5: 32.3	Number of pets, home size, use of wood stove, no of times the stove was stoked
**Mills et al. 2012 [26]**	54 households (house, bungalow, flats with/without shared entry)	Aberdeen; UK	SHS	PM2.5	Living or room in which participants spent most time in	Not reported	PM2.5 GM: 22 μg/m³	Household smoking restrictions
**Mohammadyan and Ashmore 2005 [19]**	40 households	Bradford; UK	Not reported	PM2.5	Not applicable to this study	Summer and winter	GM concentration PM2.5: 19.0 μg/m³	Air exchange rate, temperature, natural ventilation
**Morawska et al. 2011 [38]**	167 households	Vientiane, Bolikhamxay; Lao PDR	Cooking and heating (wood), smoking, soil floor	PM10, CO, NO2	Living area	December 2005–April 2006	Mean concentrations in Bolikhamxay: PM10: 1183 μg/m³ CO: 0.49 ppm NO2: 561 μg/m³ Mean concentrations in Vientiane: PM10: 1275 μg/m³ CO: 0.43 ppm NO2: 1210 μg/m³	Smoking, presence of chimney, resuspension of dust from soil floors, building materials, cooking fuel type, season
**Mullen et al. 2016 [60]**	352 homes	California; USA	Gas appliances	CO, NO2, NOx, formaldehyde, acetaldehyde	Kitchen, bedrooms	November 2011–April 2012, October 2012–March 2013	CO (ppm) kitchen highest 8-h: 3.4 kitchen highest 1-h: 6.4 NO2 (ppb) kitchen: 23 bedroom: 18 NOx (ppb) kitchen: 73 bedroom: 65 Formaldehyde (ppb) kitchen: 17 bedroom: 17 Acetaldehyde (ppb)	Gas appliance type, cooking with gas (amount), presence of pilot light, ventilation, home size

							kitchen: 9.7<br>bedroom: 9.7	
**Nasir and Colbeck 2013 [155]**	11 households (single room in shared multistorey accommodation, single bedroom flats in 3 storey buildings, 2 or 3 bedroom houses)	Colchester; UK	Smoking, cooking, heating type, cleaning, movement	PM10, PM2.5, PM1	Living room, kitchen	2004–2008	Mean 24 h concentrations (μg/m^3) in kitchen type I, II, III residences:<br>PM10: 63, 59, 30<br>PM2.5: 56, 46, 10<br>PM1: 51, 37, 5<br>PM10–PM2.5: 7, 13, 20	House size and relative occupancy, open house layout, smoking, wood burning, cooking, cleaning, ventilation, outdoor sources, season
**Nazariah et al. 2013 [149]**	212 households (villa, apartment, single storey terrace, double storey terrace, village)	Klang Valley; Malaysia	Cooking, cleaning, housework, occupant's activities, smoking	PM2.5, PM10	Living room	Not recorded	Urban mean concentrations (μg/m^3):<br>PM2.5: 50.77<br>PM10: 80.07<br>Rural mean concentrations (μg/m^3):<br>PM2.5: 25.63<br>PM10: 45.38	Location, outdoor sources, frequency of cooking, occupants activities
**Ohura et al. 2002 [111]**	41 households	Fuji; Japan	Smoking in one house	39 PAHs	Not recorded	Summer 1999, winter 1999–2000	GM concentration naphthalene: 1.5 μg/m^3	Natural ventilation, attached garage, tatami mat, building age, heating/cooling mode
**Olsen et al. 2014 [48]**	60 non-smoking households	Copenhagen; Denmark	Not reported	PNC (10–300 nm), PM2.5	Living room	February–May 2013	Median<br>PNC: 8400 #/cm3<br>PM2.5: 12.2 μg/m^3	Not reported
**Osman et al. 2007 [54]**	148 households	Aberdeen; UK	Smoking, heating	PM2.5, NO2	Living room and bedroom	October 2004–May 2005	Median concentrations:<br>PM2.5: 18 μg/m^3<br>NO2: 7.8 ppb<br>endotoxins: 95.8 EU/mg	Smoking, central heating, kitchen adjoining living room, floor area
**Park and Ikeda 2006 [88]**	1417 households	Japan—widely distributed across the country	Building materials	VOCs	Living room, bedroom	July 2000–October 2000; July 2001–September 2001	Mean concentrations (μg/m^3) new/older homes (years 1–3):<br>formaldehyde: 111/89<br>toluene: 18.7/12.0<br>p-xylene: 19.7/16.0<br>ethylbenzene: 13.7/6.3	Building materials, wooden materials, type of room, building age

							styrene: 25.3/6.0<br>limonene: 32.0/35.3<br>α-Pinene: 133.3/33.0	
**Paulin et al. 2014 [129]**	100 homes	Baltimore; USA	Cooking stoves, pilot lights (gas appliances)	NO2	Kitchen and main bedroom	June 2009–March 2011	NO2 median concentrations (ppb):<br>Kitchen:<br>baseline 12.2<br>1 week 25.5<br>3 months 24.7<br>Bedroom:<br>baseline 13.1<br>1 week 14.2<br>3 months 18.2	Using gas appliances, 3 interventions (stove replacement, ventilation hood, air purifier)
**Paulin et al. 2017 [67]**	30 households (houses, apartments, others)	Baltimore; USA	Gas cookers	NO2	Kitchen	November–March	NO2 mean concentration: 109 μg/m^3	Frequency of daily cooking appliance use, windows open for more than 10 minutes, distance from curb to front door, season
**Pavilonis et al. 2013 [140]**	197 households (single-family home, trailer)	Keokuk county; USA	Smoking, cooking, heating, pets	PM10, PM2.5, CO, CO2	Main living area	2007–2011 (all seasons)	GM concentrations (μg/m^3):<br>PM10: 21.2<br>PM2.5: 12.2<br>Endotoxins: 0.21	Use of gas appliances and AC, indoor relative humidity, overall home cleanliness, pets, smoking, outdoor concentrations, location of dwellings (rural/town), season
**Phillips et al. 2005 [81]**	42 households (detached, single-family houses)	Oklahoma city, Tulso, Ponca city, Stillwater; USA	Cleaning products, scented candles and potpourri, glues, paints, and sealants	11 VOCs	Main living area, usually kitchen or living room	Multi season	Median concentrations (μg/m^3) day, night:<br>toluene: 12, 22<br>benzene: 0.62, 1.2<br>o-xylene: 0.30, 0.67<br>p-xylene: 2.3, 3.2	Building materials, building age, building airtightness

**Pickett and Bell 2011 [119]**	10 households (single-family detached dwelling, townhouse or duplex, multiple story apartment building)	Connecticut, New York, Vermont, Massachusettes; USA	Heating fuel, fireplaces, gas stoves, attached garage, mice, indoor pesticides, pets, aromatic candles, smoking, room deodorizers, air purifiers, renovations within 6 months	CO, CO2, PM0.5, TVOCs	Room where infant spends most time	June 2009–August 2009	Mean concentrations: CO: 0.85 ppm CO2: 663.2 ppm PM0.5: 18.7 μg/m³ TVOCs: 1626 μg/m³	Building age, use of gas stove, presence of pets, remodelling the nursery, distance to nearest road
**Raaschou-Nielsen et al. 2011 [139]**	389 households	Copenhagen; Denmark	Smoking, stove candles, cooking, fireplace, cleaning	PM2.5, black smoke	Infant's bedroom	All four seasons	Mean concentrations: PM2.5: 19 μg/m³ black smoke: 0.97×10⁻⁵ m⁻¹	Smoking, renovations, season, inner city residence, traffic intensity, frying, use of an oven, toasting, burning candles, leaving windows open, vacuum cleaning
**Rancière et al. 2011 [105]**	196 homes (mainly apartments average 70 m² surface area)	Paris and surrounds; France	Smoking, floor or wall covering, cooking, heating, air fresheners	Formaldehyde, acetaldehyde, hexaldehyde, styrene, nicotine, NO2	Infant's bedroom	October–March; April–September (twice per home between 2003 and 2006)	Mean concentrations (μg/m³) cold, warm seasons: formaldehyde: 17.2, 20.9 acetaldehyde: 8.9, 8.7 NO2: 22.2, 24.8	Season, ozone, pressed wood products, nicotine levels, use of air fresheners, type and age of wall/floor coverings, cooking, heating/cooling mode
**Raw et al. 2004 [101]**	876 households (flats/bedsits, terraced, semidetached, detached and bungalows)	England; UK	Gas cooking, heating, building materials, paintings, smoking	CO, NO2, VOCs	Kitchen, bedroom	October 1997–February 1999	GM concentrations: CO (mg/m³): bedroom: 0.39 kitchen: 0.47 NO2 (μg/m³): bedroom: 11.9 kitchen: 21.8 TVOC: 210 μg/m³ formaldehyde: 22.2 μg/m³	Gas cooking, tobacco smoking, use of combustion appliances for heating, season, cooking fuel, heating fuel, presence/location

							Benzene: 3.0 μg/m³<br>m,p-xylene: 3.8 μg/m³<br>toluene: 15.1 μg/m³<br>limonene: 6.2 μg/m³	of garage, dwelling type, building age, flooring
**Rojas-Bracho et al. 2000 [125]**	18 households	Boston; USA	Not recorded	PM2.5, PM10, PM2.5–10	Main activity room of the house (excluding kitchen)	Winter, February 1996–March 1996; summer, June 1996–September 1996; winter, January 1997–February 1997	Mean concentrations (μg/m³):<br>PM2.5: 17.5<br>PM10: 31.9<br>PM2.5–10: 14.5	Air exchange rates, season, personal and outdoor concentrations
**Rojas-Bracho et al. 2004 [21]**	18 households	Boston; USA	Cleaning, cooking	PM2.5, PM10, PM2.5–10	Main activity room (excluding kitchen)	Winter and summer, 1996 and 1997	Mean concentrations (μg/m³) winter, summer:<br>PM2.5: 17.2, 17.7<br>PM10: 37.3, 28.3<br>PM2.5–10: 20.1, 10.7	Air exchange rates, ventilation, cleaning
**Romagnoli et al. 2016 [136]**	2 households in medium sized buildings	Rome; Italy	Smoking, cooking, heating, cleaning products	PM2.5, PAHs, O2, SO2, O3	Bedroom (one dwelling), dining room, balcony	March 2013	Mean concentrations (μg/m³):<br>PM2.5: 31<br>ΣPAH: 3.15	Ventilation, smoking, condensation of vapours
**Rosen et al. 2015 [22]**	27 households	Israel	Tobacco smoking, cooking, heating, outdoor sources	PM2.5	Central area	March 2013–September 2013	PM2.5 mean concentration: 0.022 μg/m³	Presence of tobacco smoke, open plan kitchen, window opening
**Rotko et al. 2000 [160]**	201 households	Helsinki; Finland	Smoking	PM2.5	Not recorded	October 1996–December 1997	PM2.5 mean concentration: 11.73 μg/m³	Not reported
**Rovira et al. 2016 [104]**	10 households	Tarragona County, Catalonia; Spain	Building materials, paints, varnishes, household cleaning products, smoking	Formaldehyde	Bedroom, living room	January 2014–February 2014	Mean concentrations (μg/m³) formaldehyde:<br>bedroom: 27.3<br>living room: 22.5	Diffusion between rooms
**Rumchev et al. 2004 [74]**	192 households	Perth; Australia	Smoking, heating, cooking, floor adhesives, furnishings, polishes, room fresheners, cleaning, solvents, paint	10 VOCs	Living room	Winter, June 1998–September 1998; summer, December 1998–March 1999	Median concentrations (μg/m³) cases vs. control:<br>benzene: 24.8 vs. 11.8<br>toluene: 11.9 vs. 6.2<br>m-xylene: 1.4 vs. 0.7<br>o,p-xylene: 5.9 vs. 3.2<br>ethylbenzene: 1.4 vs. 0.8<br>TVOCs: 78.5 vs. 36.6	Recently painted houses, smoking, new carpet or furnishing, dust mite allergen

**Russo et al. 2015 [156]**	32 households (15 smoking vs. 17 non-smoking)	Boston; USA	Smoking, cooking, candles	PM2.5	Primary living area; common entries or hallway	August–December 2012	PM2.5 median (μg/m^3)<br>resident smokers: 10.6<br>hallway/shared entry: 5.1<br>smoke-free buildings: 4.8<br>smoking-permitted buildings: 8.1	Smoke transfer, open windows
**Ryan et al. 2015 [16]**	168 households	California, Texas, New Jersey; USA	Flooring, heating, use of household and personal care products, cooking, candles, road traffic, industrial emissions	PM2.5	Not reported	Not reported	PM2.5 mean concentration: 18.5 μg/m^3	Presence of a basement, unvented appliances, proximity to industry, service stations or restaurants, presence and type of air conditioning, attached garage
**Saijo et al., 2004 [83]**	96 (mainly detached wooden houses also some duplexes)	Sapporo; Japan	Building materials	VOCs	Living room	August 2001–September 2001	GM concentrations:<br>formaldehyde: 56.0 ppb<br>acetaldehyde: 10.2 ppb<br>ethylbenzene: 18.7 μg/m^3<br>toluene: 325.5 μg/m^3<br>limonene: 25.0 μg/m^3<br>xylene: 26.0 μg/m^3<br>TVOCs: 482.6 μg/m^3	Ventilation, dampness, presence of pets, household size, smoking, building age
**Saraga et al. 2010 [85]**	2 households (apartments)	Athens; Greece	Smoking	PM1, PM2.5, benzene, toluene, m.p-xylene, o-xylene	Living room	May 2005	Mean concentrations (μg/m^3)<br>smokers vs. non-smokers:<br>benzene: 6.85 vs. 5.06<br>m,p-xylene: 8.14 vs. 9.63<br>toluene: 23.3 vs. 18.6<br>o-xylene: 4.25 vs. 3.56	Outdoor air, smoking, ventilation, air exchange rate, temperature, wind speed, relative humidity of outdoor air
**Schneider et al. 2001 [86]**	405 households	Ertfurt, Hamburg; Germany	Oil stoves, paint, solvents, cooking, cleaning, heating	VOCs	Living room, bedroom	June 1995–November 1996	Median concentrations (μg/m^3)<br>Hamburg vs. Erfurt:<br>benzene: 1.48 vs. 2.17<br>toluene: 20.46 vs. 37.29<br>ethylbenzene: 0.70 vs. 1.67<br>m,p-xylene: 2.92 vs. 4.17<br>o-xylene– 0.79 vs. 1.20	Seasons, insulation, proximity to busy road, ventilation, location (urban/rural)

**Simoni et al. 2004 [123]**	421 households	Po Delt, Pisa; Italy	Gas furnace, gas water furnace, carpets, ETS, wood burning, smoking	PM2.5, NO2	Kitchen, living room, bedroom	Winter and summer Po Delta 1991–1992 and Pisa 1993–1994	NO2 mean concentrations (ppb): winter: 18.5 summer: 14	Type of heating system, location (urban/rural), ventilation
**Simons et al. 2007 [151]**	120 households (detached or duplex homes in the suburbs and inner city)	Baltimore; USA	Gas stove, smoking, disrepair, cockroach or mice infestation, pets, mould	PM 2.5, PM10, O3, NO2	Child's bedroom	Unknown	Mean concentrations (μg/m^3): PM10: 23 PM2.5: 12 O3: 0.03 NO2 < detection limit	Smoking, gas stoves, carpet, pets, moisture, mildew, crac+A85:I85ks in walls, leaky roof, temperature, musty smell, natural ventilation
**Singleton et al. 2017 [92]**	63 households	Alaska; USA	Wood stove, fuel oil, visible mould, tobacco smoke, detergent, insecticide, fuel presence/ storage	PM2.5, VOC, CO2	Living room	Season not reported, 2012–2015	Median concentrations: PM2.5: 33 μg/m^3 TVOC: 99.3 μg/m^3 CO2: 1401 ppm	Crowding, reduced ventilation, use of homes as workshops
**Su et al. 2013 [147]**	310 households	Elizabeth, Houston, Los Angeles; USA	Cooking, heating, ventilation, cleaning devices, paint and solvents, pets	18 VOCs	Not recorded	Summer 1999–spring 2001	Mean concentrations (μg/m^3): benzene: 3.54 ethylbenzene: 2.55 toluene: 15.26 m/p-xylene: 7.39 o-xylene: 2.49	Living in Houston, attached garage, self-pumped gas, wind speeds, house AERs, use of gas heating/stove, open windows/doors
**Takeda et al. 2009 [95]**	104 households (detached houses)	Sapporo City; Japan	Pets, smoking, room fragrance, insect repellent, mould	Formaldehyde, acetaldehyde, VOCs	Living room	September 2004–October 2004	Median concentrations (μg/m^3) SBS, non-SBS symptoms: formaldehyde: 70.1, 63.2 acetaldehyde: 35.9, 33.5 VOCs: 181.6, 127.6	Presence of pets, smoking, using room fragrance, using insect repellent, time spent in dwelling, building age, building materials

**Takigawa at al. 2010 [108]**	425 households (conventional homes, detached houses)	Hokkaido, Fukushima, Osaka, Okayama, Fukuoka; Japan	Wood, cleaning products, nail polish and remover	Aldehydes, VOCs	Living room	Autumn, September–December in 2004 and 2005	Median concentrations (μg/m³) SBS, non-SBS symptoms: formaldehyde: 48.5, 39.4 acetaldehyde: 24.3, 22.2 benzene: 1.1, 1.1 toluene: 12.9, 12.9 ethylbenzene: 3.2, 2.8 xylene: 6.0, 5.8	Age of building, building materials, detergents, air fresheners
**Takigawa et al. 2012 [99]**	260 households (<= 6 years old in 2003)	Japan (6 cities)	Smoking, pets, dew condensation, mould, use of moth repellent, use of air freshener	29 VOCs and 13 aldehydes	Living room	Autumn, September–December in 2004 and 2005	Median concentrations (μg/m³) in 2004, 2005: formaldehyde: 39.2, 31.5 acetaldehyde:20.8, 15.7 benzene: 1.1, 1.6 toluene: 11.8, 10.9 xylene: 5.8, 5.7 ethylbenzene: 2.7, 3.3	Not recorded
**Tanaka-Kagawa et al. 2005 [69]**	50 households	Iwate, Yamanshi, Shiga, Hyogo, Kochi, Fukuoka; Japan	Not reported	132 VOCs	Not reported	January 2005–February 2005	Mean concentrations (μg/m³): toluene: 16 m,p-xylene: 9.3 benzene: 3.2 ethylbenzene: 5.3 o-xylene: 4.1 naphthalene: 2.6 limonene: 30 α-pinene: 47 β-pinene: 19	Not reported
**Taneja et al. 2008 [40]**	20 households (10 urban and 10 roadside)	Agra; India	Fuel for cooking, cleaning substance, cooking	CO2, CO, NO2, NO, SO2, Cl2, H2S, NH3, and PM10, PAHs	Living room	October 2004–December 2005	Mean monthly concentrations rural vs. roadside sites: SO2: 10 ppb vs. 17 ppb NO2: 17.8 ppb vs. 17.1 ppb CO2: 422 ppm vs. 492 ppm CO: 1.12 ppm vs. 1.7 ppm	Cooking fuel, kerosene and gas heater use, wood coal stoves, smoking, natural ventilation, wood, heavy oils
**Topp et al. 2004 [145]**	631 households	Erfurt, Hamburg, Zerbst, Bitterfeld, Hettstedt; Germany	Not reported	BTEX, NO2	Living room, bedroom/ nursery	June 1995–May 1997; April 1996–September 1998	Median concentrations (μg/m³) living rooms at 1st and 2nd visit: NO2: 14.9, 15.2 toluene: 35.7, 32.3 m,p-xylene: 3.5, 4.0 o-xylene: 1.1, 1.1 ethylbenzene: 1.5, 1.5 benzene: 2.0, 2.5 Median concentrations (μg/m³)	Smoking, gas cooking

							bedroom/nursery at 1st and 2nd visit:<br>NO2: 13.8, 14.5<br>toluene: 21.0, 20.3<br>m,p-xylene: 3.0, 3.2<br>o-xylene: 0.7, 0.6<br>ethylbenzene: 1.2, 1.1<br>benzene: 1.7, 2.1	
**Tunno et al. 2015 [31]**	21 households	Pittsburgh; USA	Cooking, smoking, cleaning, outdoor sources	PM2.5	Main activity room	Summer, July 2011–September 2011; winter, January 2012–March 2012	PM2.5 mean concentrations (μg/m^3):<br>summer: 25.8<br>winter: 18.9	Windows opening, cooking activity, cigarettes smoked, kitchen cleaning, number of children
**Uchiyama et al. 2015 [55]**	602 households	Japan	Household products (e.g., moth balls), space deodorizers, insecticides, kerosene heaters, gas stoves and heaters, pets, building materials	Carbonyls, VOCs, NO2, O3	Living room	Winter 2012, 2013, 2014 (January–March), summer 2012, 2013 (July–September)	Benzene (μg/m^3):<br>winter: 2.3<br>summer: 1.3<br>Toluene (μg/m^3):<br>winter: 11<br>summer: 12<br>NO2 (μg/m^3):<br>winter: 220<br>summer: 13<br>Formaldehyde (μg/m^3):<br>winter: 13<br>summer: 34<br>Acetaldehyde (μg/m^3):<br>winter: 22<br>summer: 17	Season/temperature, drinking alcohol (acetaldehyde)
**Vanker et al. 2015 [143]**	600 homes	Drakenstain, Western Cape; South Africa	Heating, cooking, smoking, pesticides, cleaning products	PM10, SO2, NO2, CO, VOCs	Communal area/main living room	March 2011–May 2014	Median concentrations (μg/m^3):<br>PM10: 33.1<br>CO/SO2: 0<br>NO2: 7.9<br>benzene: 5.6<br>toluene: 19.8	Type of home/kitchen, ventilation, informal construction, crowding, fuel usage
**Villanueva et al. 2015 [76]**	22 households	Puertollano; Spain	Smoking, furniture, furnishings, heating, cooling	VOCs Carbonyls	Living room	May–June 2011	Mean concentrations (μg/m^3):<br>benzene: 1.9<br>toluene: 12.0<br>ethylbenzene: 3.4<br>m/p-xylene: 7.0<br>formaldehyde: 54.6	Ventilation, smoking, furniture age, use of heating, air conditioning, indoor carpet

							acetaldehyde: 23.0<br>α-pinene: 18.5<br>limonene: 17.1	(toluene), location
**Wallace et al. 2003 [20]**	294 households	Boston, Bronx, Chicago, Dallas, Manhattan, Seattle, Tucson; USA	Smoking, cooking, incense, cleaning	PM2.5	Living area if possible–alternatives including child's bedroom or dining room	Unspecified	PM2.5 mean concentration: 27.7 µg/m^3	Smoking, cooking, type of cooking (frying), burning incense, housing type (apartment housing)
**Wallace et al. 2006 [44]**	1 townhouse (3 level with 4 bedrooms)	Reston; USA	Cooking appliances, candles, gas clothes dryer, air fresheners	UFP	Basement/ recreation room	October 1996 and March of 2001	Mean concentrations (#/cm^{-3}) for six size ranges of particles:<br>10–18: 4589<br>18–50: 10101<br>50–100: 6045<br>100–200: 2238<br>200–450: 405<br>450–950: 321	Use of central fan/duct system, type of cooking, burning incense and candles, natural ventilation
**Wang et al. 2017 [90]**	25 households (19 homes in London and 6 homes in York)	London and York; UK	Cleaning products, consumer products (air fresheners, fragrance, scented candles)	VOCs, Formaldehyde	Living room	Spring, 2015; Autumn, 2015	Range of 5 day means (µg/m^3):<br>London:<br>VOC <LOD–54<br>York:<br>α-pinene: 2–229<br>d-limonene: 18–1439<br>isoprene: 11–22<br>benzene 7–19<br>formaldehyde (72 hour average in 3 homes): 46.8 µg/m^3	Single or double glazed windows, open plan kitchen, occupants activities, e.g., cleaning, use of personal care products
**Weichenthal et al. 2007 [47]**	36 households (single-family homes, town houses)	Montreal, Pembroke; Canada	Heating, smoking, vacuuming, dusting, kitchen exhaust, candles, gas dryer	UFP, PM4	Kitchen and living room	December 2005–March 2006	UFP mean concentrations by heating system type (#/cm3):<br>electric board: 17064<br>wood stove: 17546<br>forced air oil furnace: 11039<br>forced air gas furnace: 13009<br>PM4 mean concentration: 192.07 µg/m^3	Cooking time, heating system type, occupant density, number of smokers, urban location, age of the building, electric heaters, size of the building, cleaning frequency, type

								of cooking system
**Weitzman et al. 2016 [28]**	33 households (11 homes where only hookah was smoked, 12 homes where only cigarettes were smoked, and 10 homes where neither hookahs nor cigarettes were smoked)	Dubai; UAE	Hookahs, cigarettes, incense	PM2.5, black carbon, elemental and organic carbon, CO	Room where smoking occurred and in one adjacent non-smoking room in homes where hookahs or cigarettes were smoked. In non-smoking homes, bedroom and living room (averaged values)	Not reported	Mean concentrations PM2.5 (μg/m^3): hookah/adjacent 428.6/210.8 cigarette/adjacent 201.2/96.7 non-smoking 93.2 BC (μg/m^3): hookah/adjacent 5.4/3.7 cigarette/adjacent 4.2/3.0 non-smoking 2.1 CO (mg/m^3): hookah/adjacent 12.6/6.7 cigarette/adjacent 2.6/1.8 non-smoking 1.7	Use of hookahs or cigarettes at home, length of smoking time, the number of smokers, number of hookahs or cigarettes smoked, ventilation during smoking sessions, number of children in households, size of the room
**Wheeler et al. 2013 [70]**	3857 households (single detached, double, row/terrace, duplex, low-rise or high-rise apartment)	Canada	Renovations, furnishings, heating source, household, cleaning and personal care products, crafts and DIY (products), pesticides, candles, smoking	BTEX: benzene, toluene, ethylbenzene, m/p-xylenes, o-xylene	Living or family room	August 2009–November 2011	Mean concentrations (μg/m^3): benzene: 1.95 toluene: 19.17 ethylbenzene: 4.09 m/p-xylene: 14.42 o-xylene: 4.16	Garage on property, regular smoking, renovations, No. of occupants, use of paint remover, use of fragrance, dwelling type
**Wigzell et al 2000 [30]**	10 households	Oxford; UK	Cooking, smoking	PM2.5, TSP, fine particles	Kitchen, living room	June 1999–July 1999	Mean concentrations (μg/m^3) kitchen, living room: PM2.5: 13, 12 TSP: 32, 41 fine particles: 942, 1033	Gas cooking, fan use when cooking, vacuuming, natural ventilation, smoking
**Wyss et al 2016 [23]**	36 households (14 stove users, 22 non-users)	Oslo; Norway	Wood stove or fireplace, candles, frying food, activities accidentally producing smoke	PM2.5	Living room	November 2012–February 2013	PM2.5 hourly mean concentrations (μg/m^3): homes with wood stove: 15.6 homes without stove: 12.6	Ventilation (opening windows), age of stove used

**Yeatts et al. 2012 [107]**	628 households in rural and urban areas	UAE (all seven emirates)	Incense, gas stove, smoking	SO2, NO2, H2S, formaldehyde, CO, PM2.5, PM2.5–10, PM10	Living room	October 2009–May 2010	Median concentrations:<br>SO2: <0.010 ppm<br>NO2: <0.006 ppm<br>H2S: <0.060 ppm<br>formaldehyde: 0.006 ppm<br>CO: 0.761 ppm<br>PM2.5: 6.20 $\mu g/m^3$<br>PM2.5–10: 36.95 $\mu g/m^3$<br>PM10: 43.98 $\mu g/m^3$	Dwelling type, frequency of incense use, cooking equipment type, kitchen configuration and gas cooking equipment
**Zipprich et al. 2002 [62]**	23 households	Richmond; USA	Air conditioner, stove, heating	NO2, NO, NOx	Living room or dining room and near bedroom	July 1999–September 1999	Mean concentrations (ppb) bedroom, living room:<br>NO2: 18, 19<br>NO: 57, 65<br>NOx: 75, 84	Smoking, carpet, humidity, building age

© 2020 by the authors. Licensee MDPI, Basel, Switzerland. This article is an open access article distributed under the terms and conditions of the Creative Commons Attribution (CC BY) license (http://creativecommons.org/licenses/by/4.0/).
